# Supplementary material for: Aminoacyl sulfonamide assembly in SB-203208 biosynthesis
Source: Nat Commun. 2019 Jan 14;10:184. doi: 10.1038/s41467-018-08093-x (PMC6331615; doi:10.1038/s41467-018-08093-x)
Supplement: Supplementary file 1 — Supplementary Information [file 41467_2018_8093_MOESM1_ESM.pdf]

Supplementary Information

Aminoacyl sulfonamide assembly in SB-203208 biosynthesis

Zhijuan Hu[1,2], Takayoshi Awakawa[1,3,]*, Zhongjun Ma[2], Ikuro Abe[1,3,]*

[1] Graduate School of Pharmaceutical Sciences, The University of Tokyo, 7-3-1 Hongo, Bunkyo-ku, Tokyo 113-0033, Japan

[2] Ocean College, Zhejiang University, Zhoushan, 316000, China

[3] Collaborative Research Institute for Innovative Microbiology, The University of Tokyo, Yayoi 1-1-1, Bunkyo-ku, Tokyo 113-8657, Japan

These authors contributed equally: Zhijuan Hu and Takayoshi Awakawa

*Address correspondence to awakawa@mol.f.u-tokyo.ac.jp, abei@mol.f.u-tokyo.ac.jp

Supplementary Figures

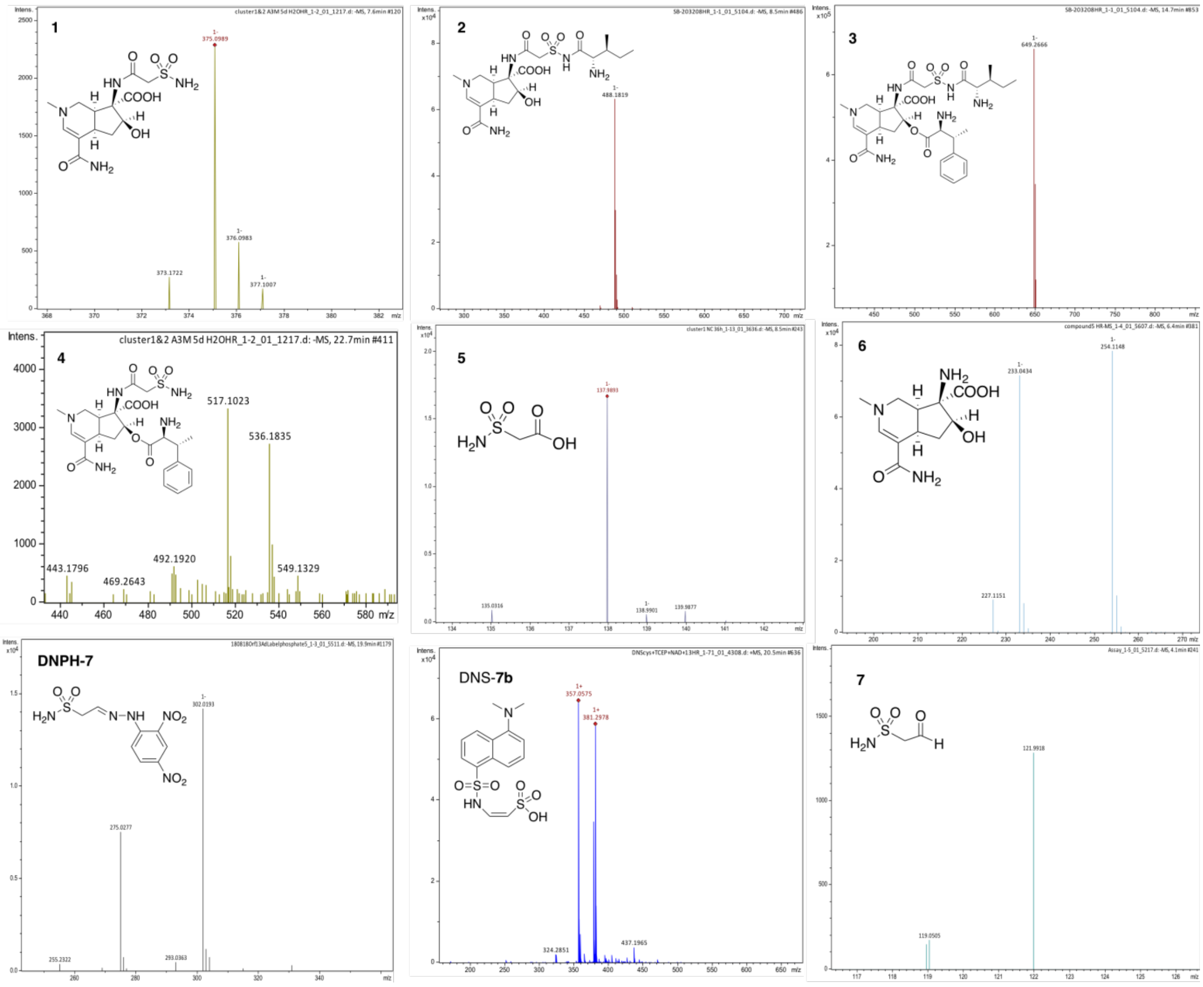

Supplementary Figure 1. HR-ESI-MS spectra of related compounds.
All MS data are detected as a negative ion $[M-H]^-$.

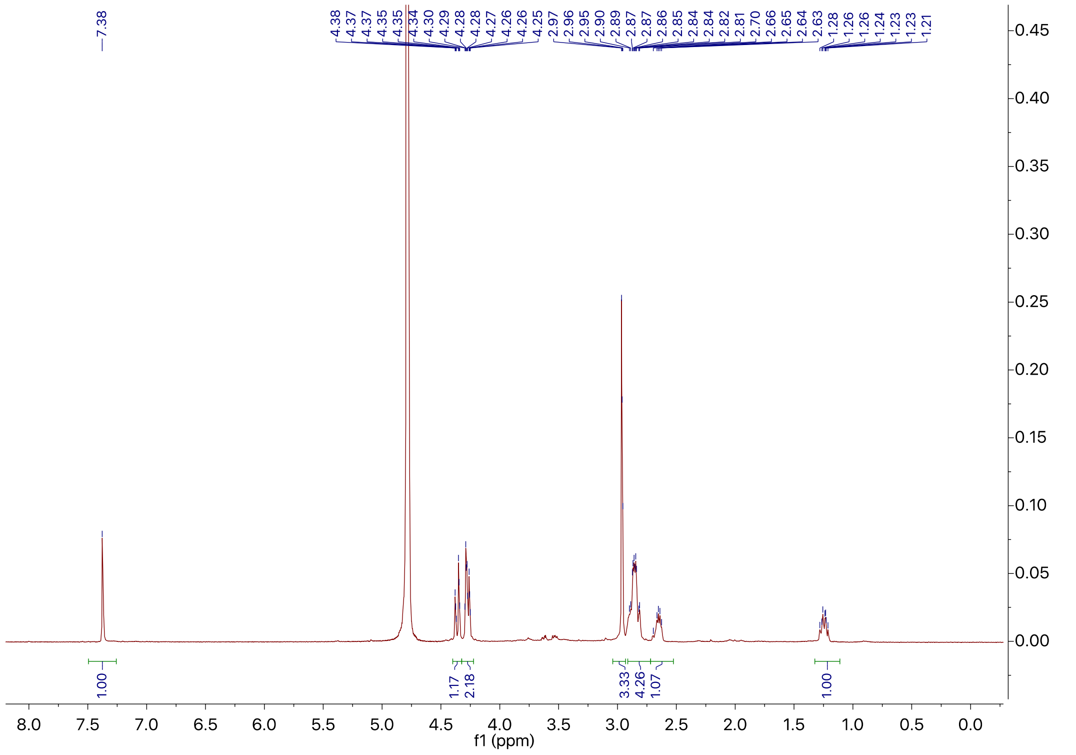

Supplementary Figure 2. ^{1}H NMR spectrum of **1** (D_2O)

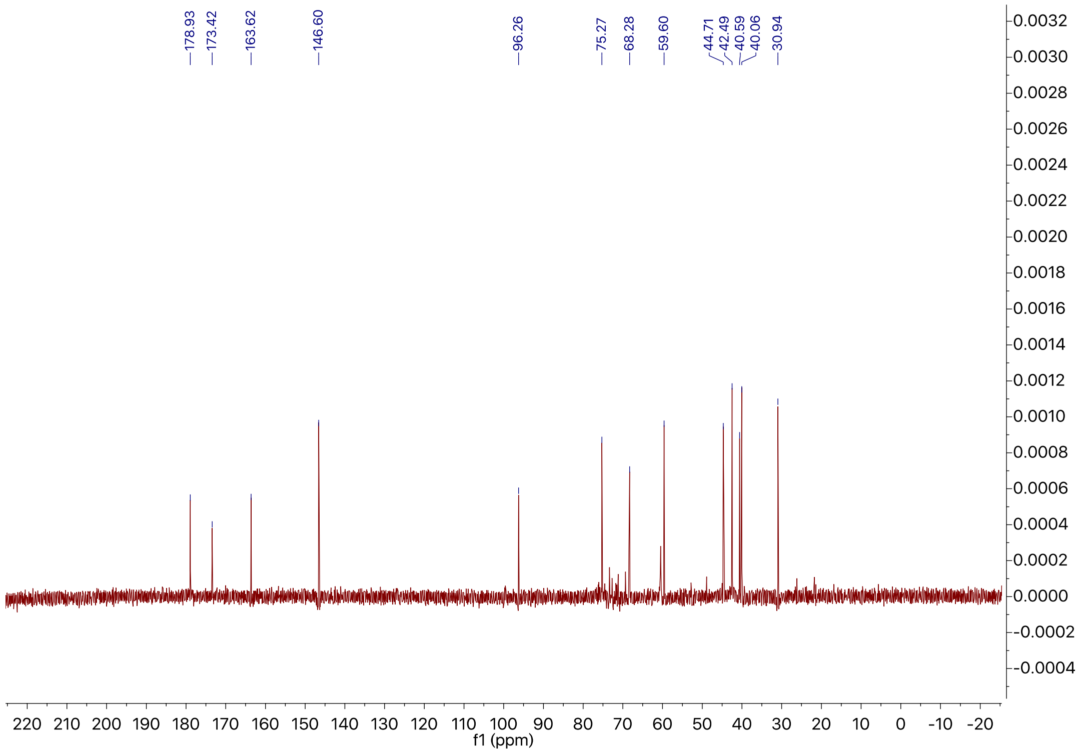

Supplementary Figure 3. ^{13}C NMR spectrum of **1** (D_2O)

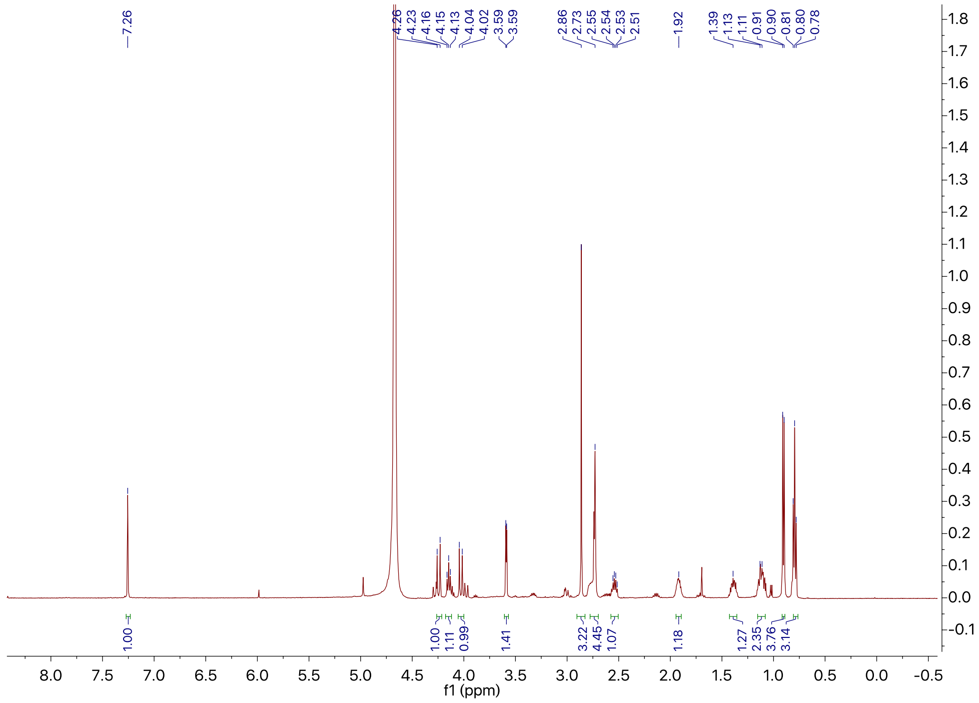

Supplementary Figure 4. ^{1}H NMR spectrum of **2** (D_2O)

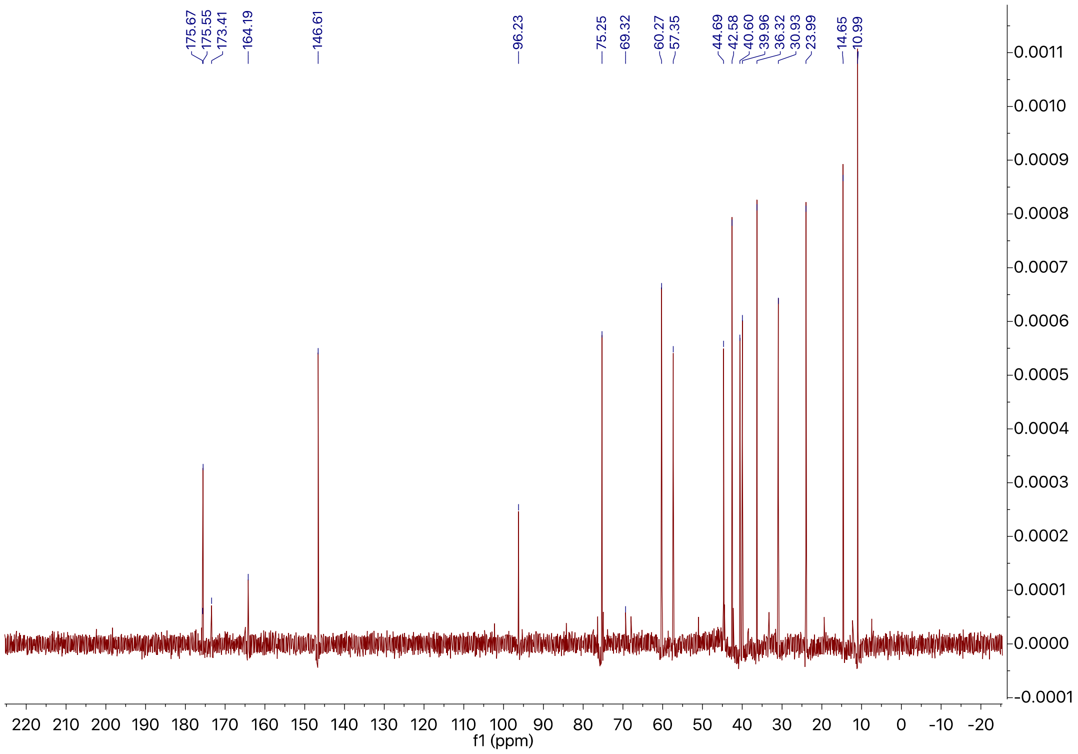

Supplementary Figure 5. ^{13}C NMR spectrum of **2** (D_2O)

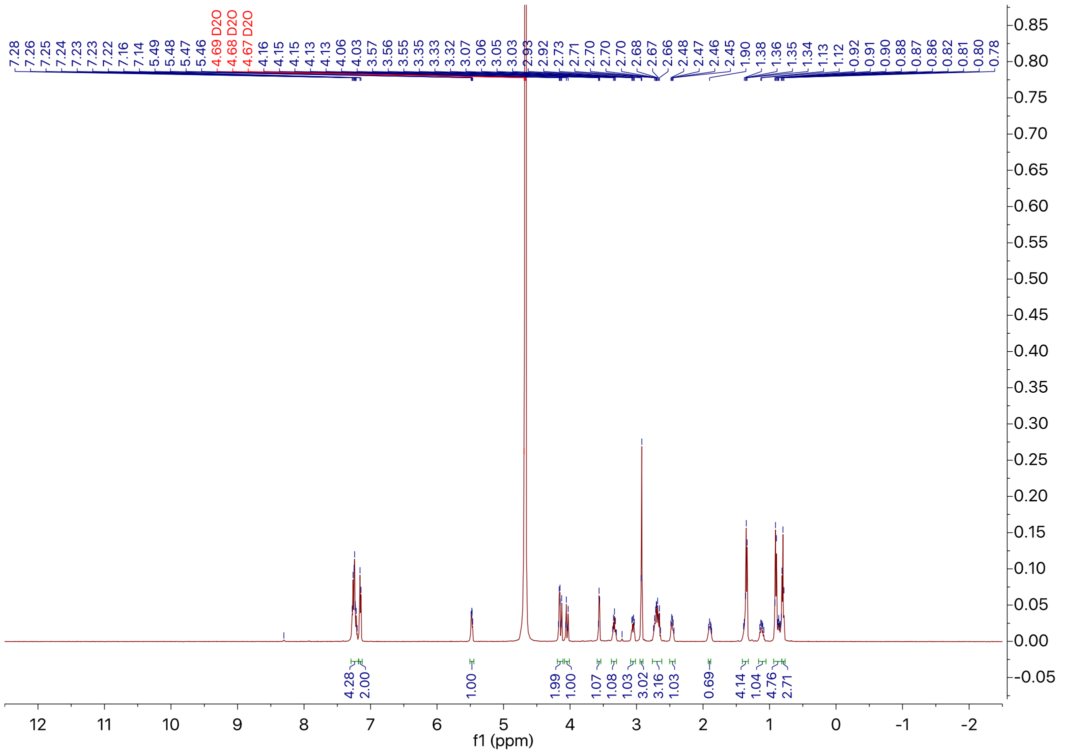

Supplementary Figure 6. ^{1}H NMR spectrum of **3** (D_2O)

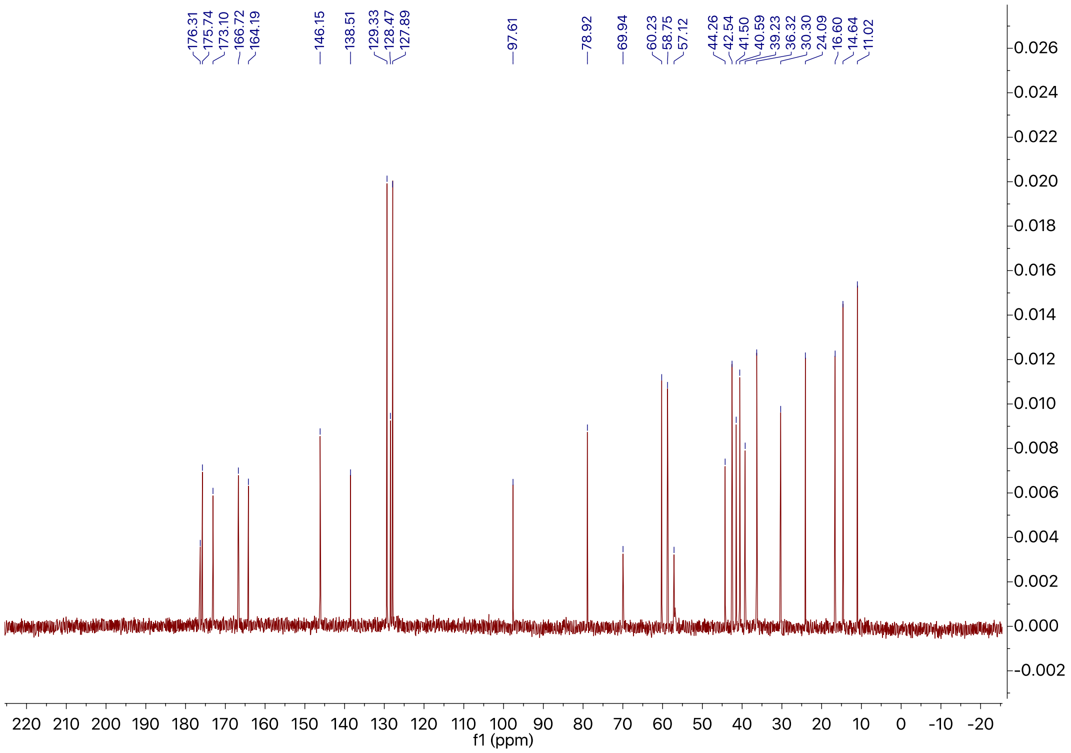

Supplementary Figure 7. ^{13}C NMR spectrum of **3** (D_2O)

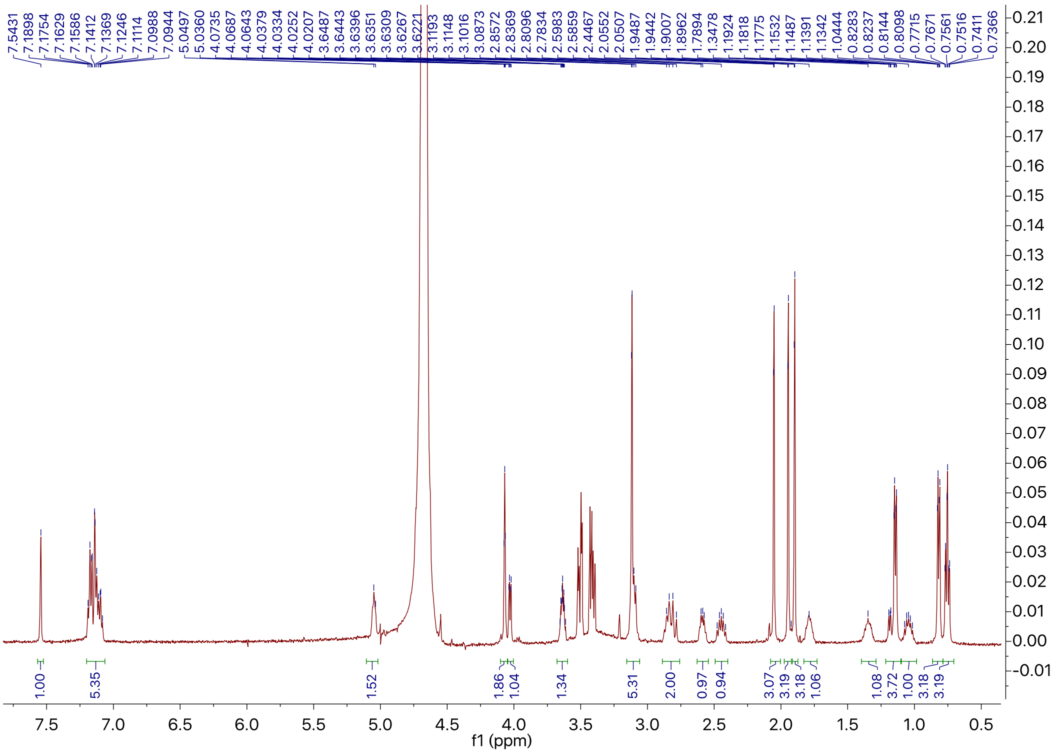

Supplementary Figure 8. ^{1}H NMR spectrum of triacetyl-**3** (D_2O)

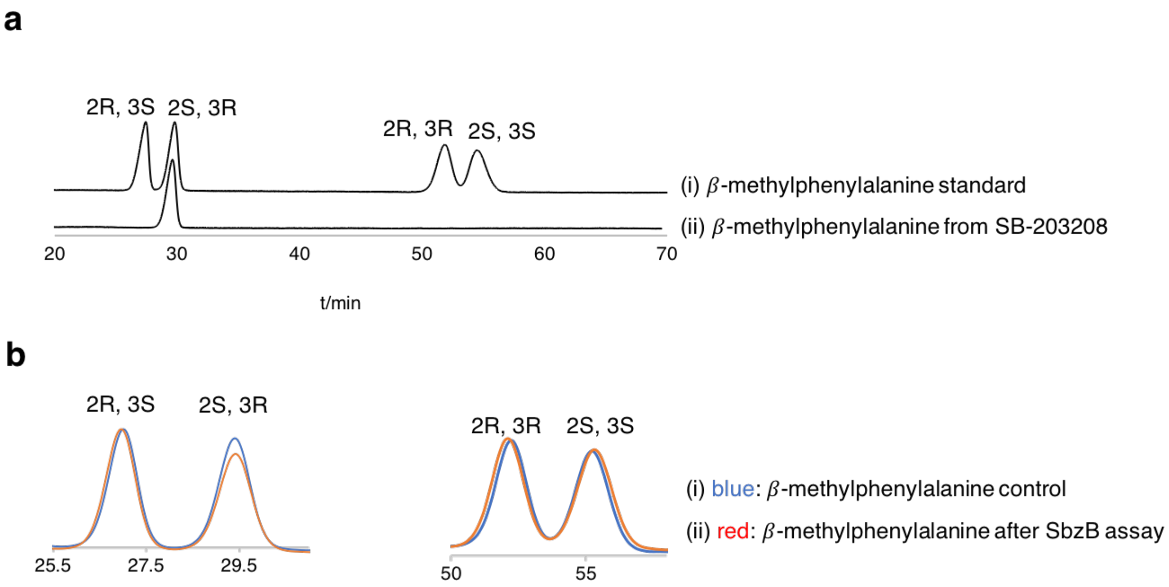

Supplementary Figure 9. Chiral HPLC analyses. **a)** (i) *β* -methylphenylalanine standard, (ii) *β*-methylphenylalanine from SB-203208 degradation; **b)** Selectivity of (*2S*, *3R*)- *β*-methylphenylalanine by SbzB adenylation assay. Monitored at 210 nm.

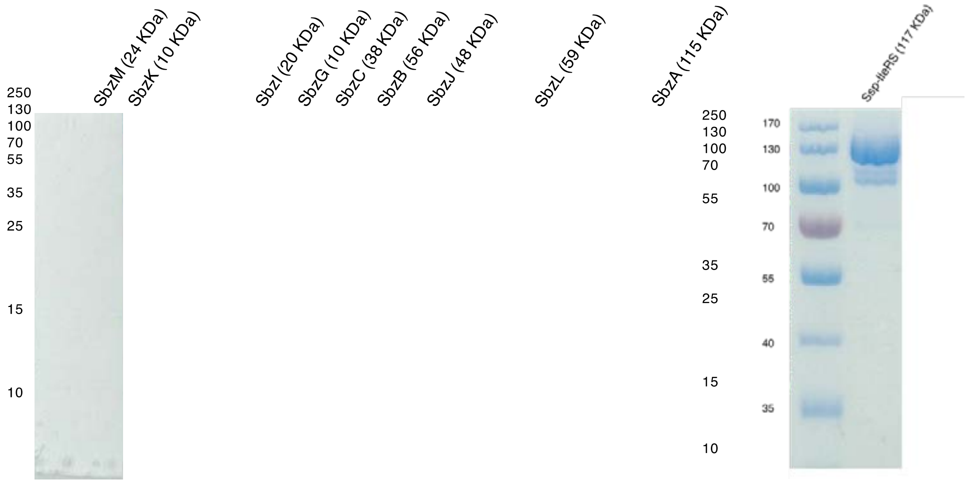

Supplementary Figure 10. SDS-PAGE of the purified proteins. SbzM (24 kDa, Lane 2), SbzK (10 kDa, Lane 3), SbzI (20 kDa, Lane 5), SbzG (10 kDa, Lane 6), SbzC (38 kDa, Lane 7), SbzB (56 kDa, Lane 8), SbzJ (48 kDa, Lane 9), SbzL (59 kDa, Lane 11), SbzA (115 kDa, Lane 14), and Ssp-IleRS (117 kDa, Lane 16). Lane 1,4,10,12,13: Thermo Scientific PageRuler Plus Prestained Protein Ladder (250, 130, 100, 70 (purple), 55, 35, 25, 15, 10 kDa), Lane 15: Thermo Scientific PageRuler Prestained Protein Ladder (170, 130, 100, 70 (purple), 55, 40, 35 kDa).

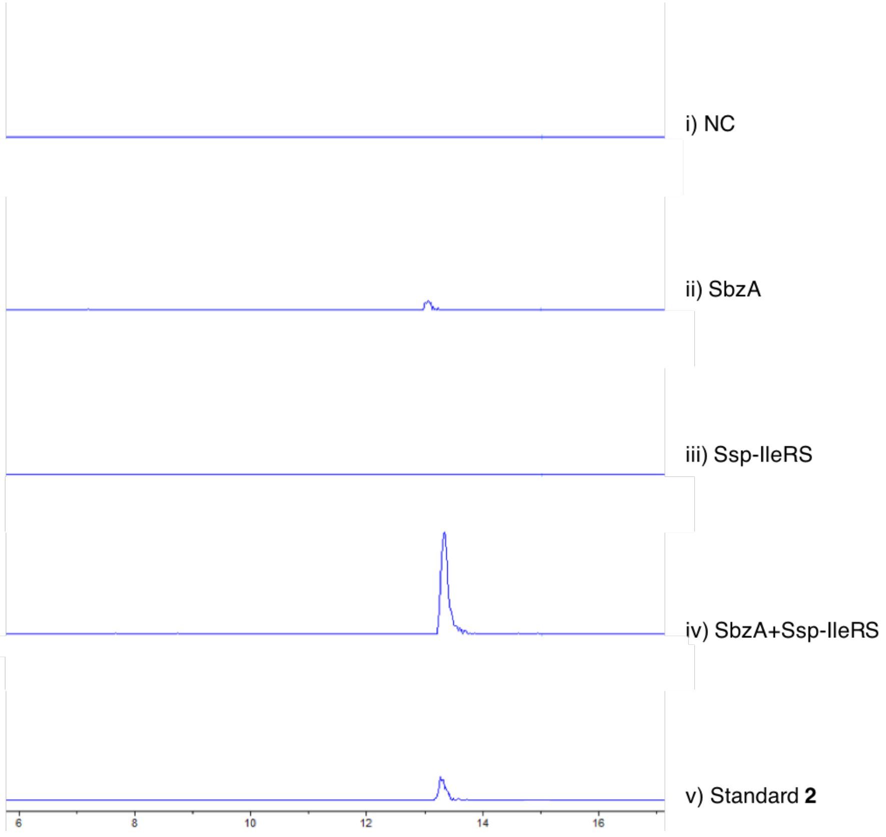

Supplementary Figure 11. LC-MS analyses of the products from the isoleucyl transfer reactions onto altemicidin (**1**). i) The reaction without any enzyme, ii) SbzA, iii) Ssp_IleRS, iv) SbzA+Ssp_IleRS, and v) authentic standard **2**. The EIC chromatogram 488.1821 which corresponds with the m/z $[M-H]^-$ of **2**.

a

b

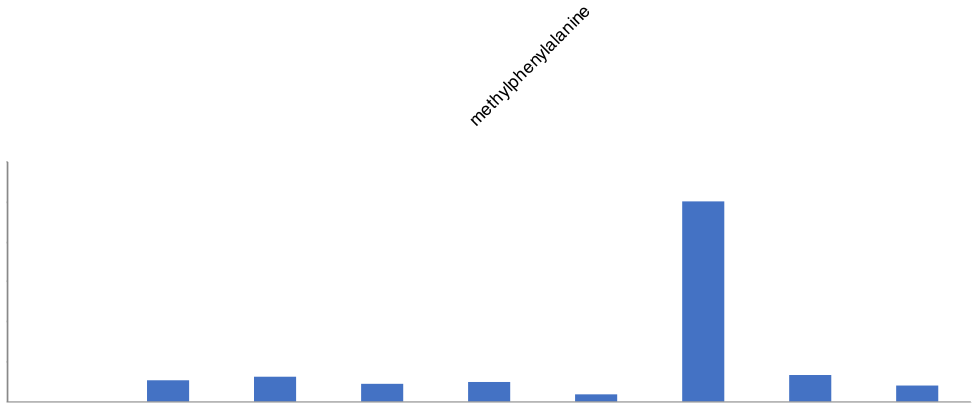

Supplementary Figure 12. Substrate specificity of AMP-ligases. a) SbzB; b) SbzL. The activity was calculated with malachite green assay, and the relative activity was calculated with comparison between the most preferred substrates (100%) and the others.

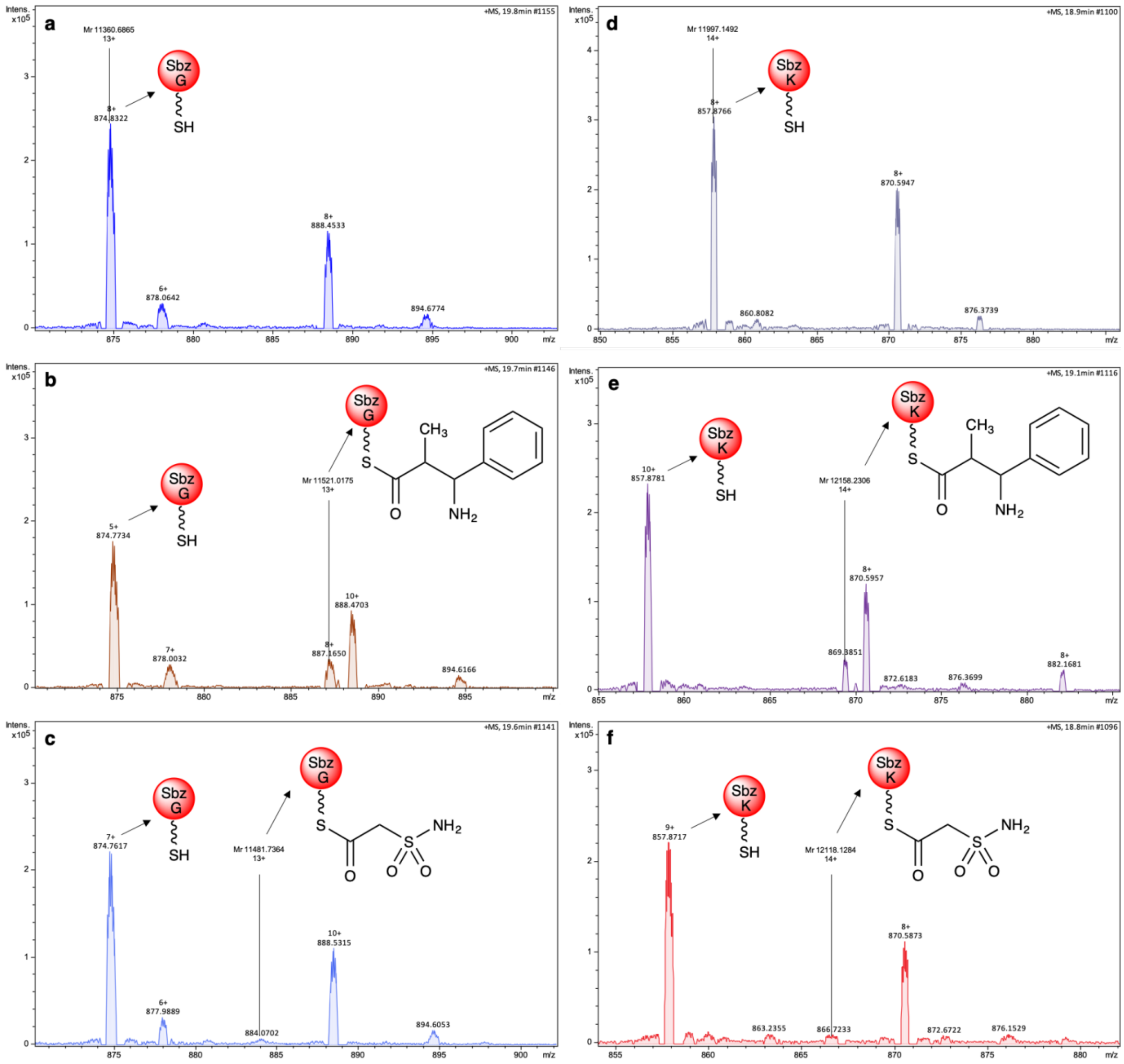

Deconvoluted ESI-MS	Exp.	Calc.
holo-SbzG	11360	11360
holo-SbzG- β-methylphenylalanine	11521	11521
holo-SbzG-2-sulfamoylacetic acid	11481	11481
holo-SbzK	11997	11997
holo-SbzK- β-methylphenylalanine	12158	12158
holo-SbzK-2-sulfamoylacetic acid	12118	12118

Supplementary Figure 13. LC-MS analysis of carrier protein. (**a**) holo-SbzG; (**b**) holo-SbzG incubated with SbzB and β-methylphenylalanine; (**c**) holo-SbzG incubated with SbzL and 2-sulfamoylacetic acid; (**d**) holo-SbzK; (**e**) holo-SbzK incubated with SbzB and β-methylphenylalanine; (**f**) holo-SbzK incubated with SbzL and 2-sulfamoylacetic acid. Deconvoluted ESI-MS was summarized.

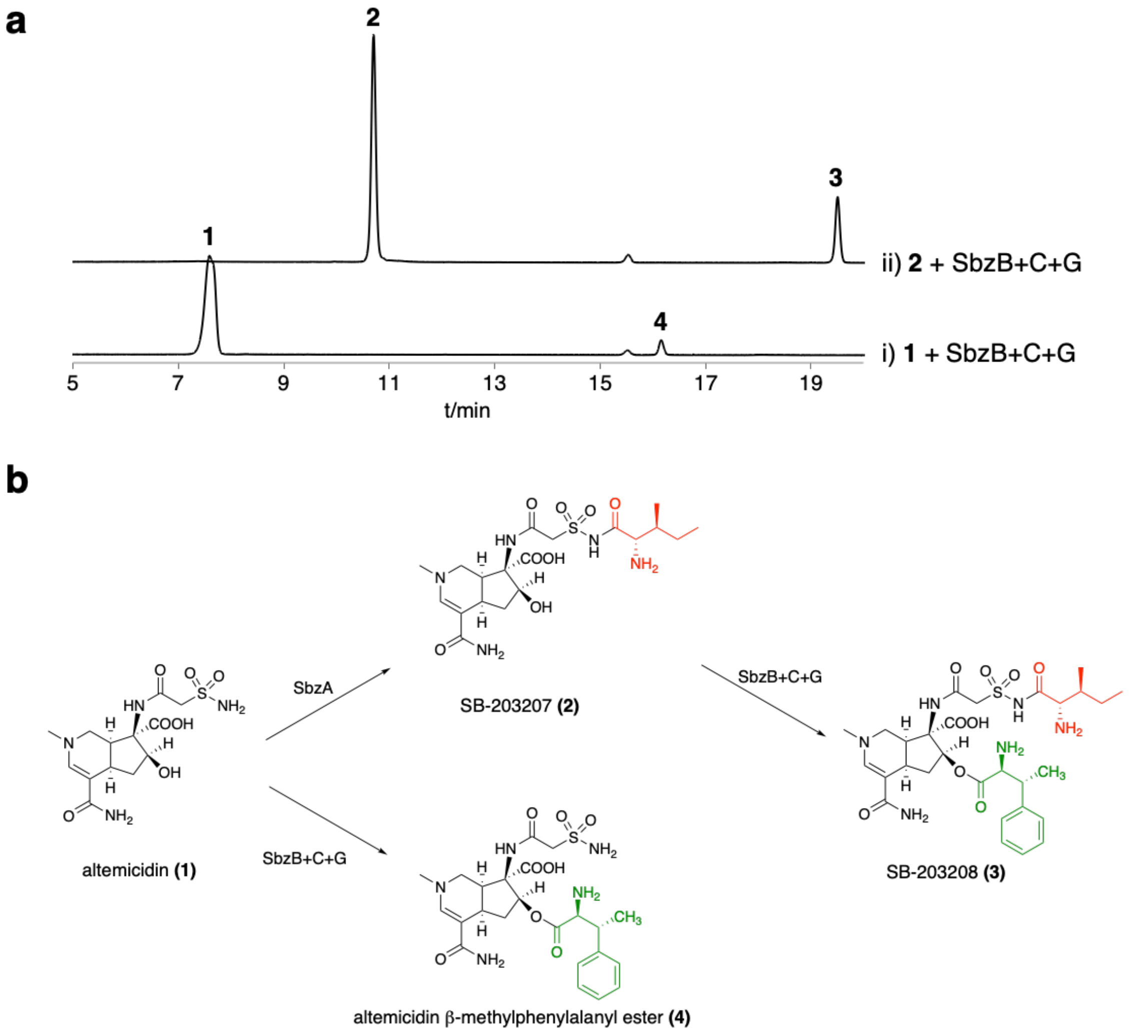

Supplementary Figure 14. The investigation of the substrate specificity of SbzBCG reactions. The HPLC analysis of the reaction by using **1** or **2** as substrate (A, i or ii, monitored at 300 nm) and their reaction scheme (B).

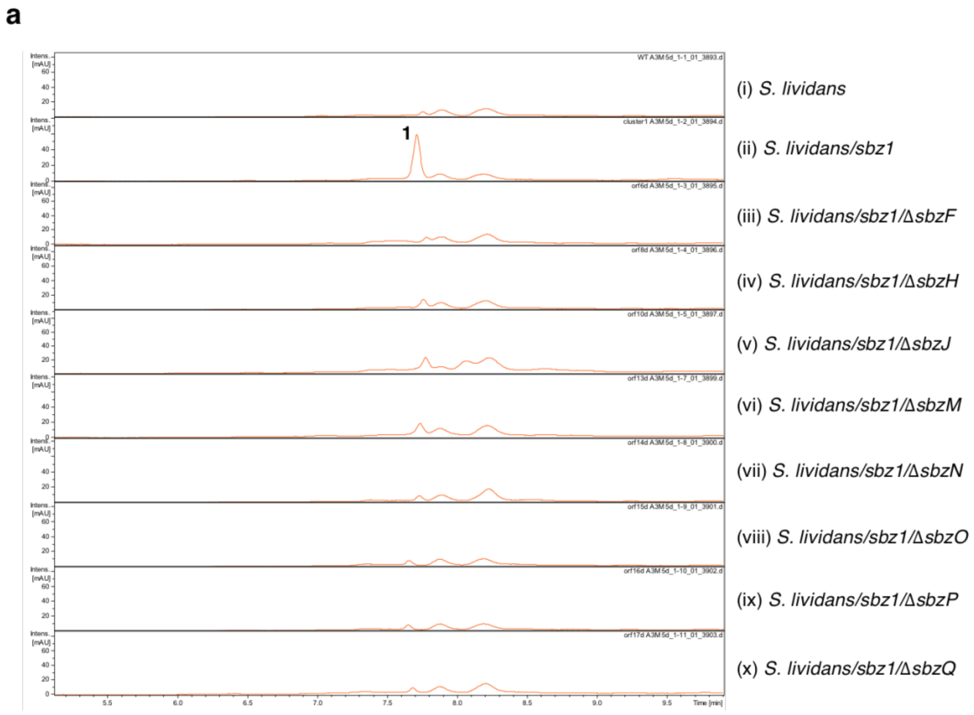

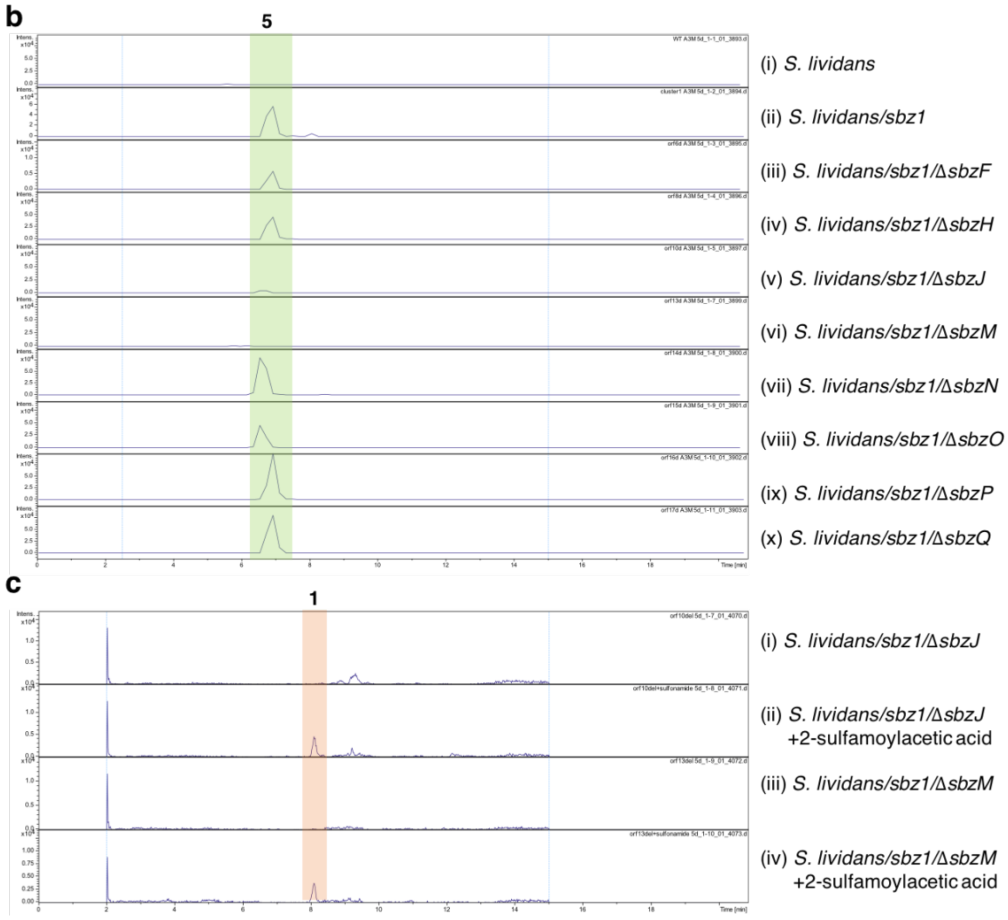

Supplementary Figure 15. Verification of the genes related to biosynthesis of **5**. **a**) UV chromatogram of the water extract of the gene deletion strains. Detected at 300 nm, **b**) EIC of **5** ([M-H]$^-$ m/z 137.9867) in gene deletion strains; **c**) EIC of **1** ([M-H]$^-$ m/z 375.0980) in gene deletion strains and its complementation of **5**.

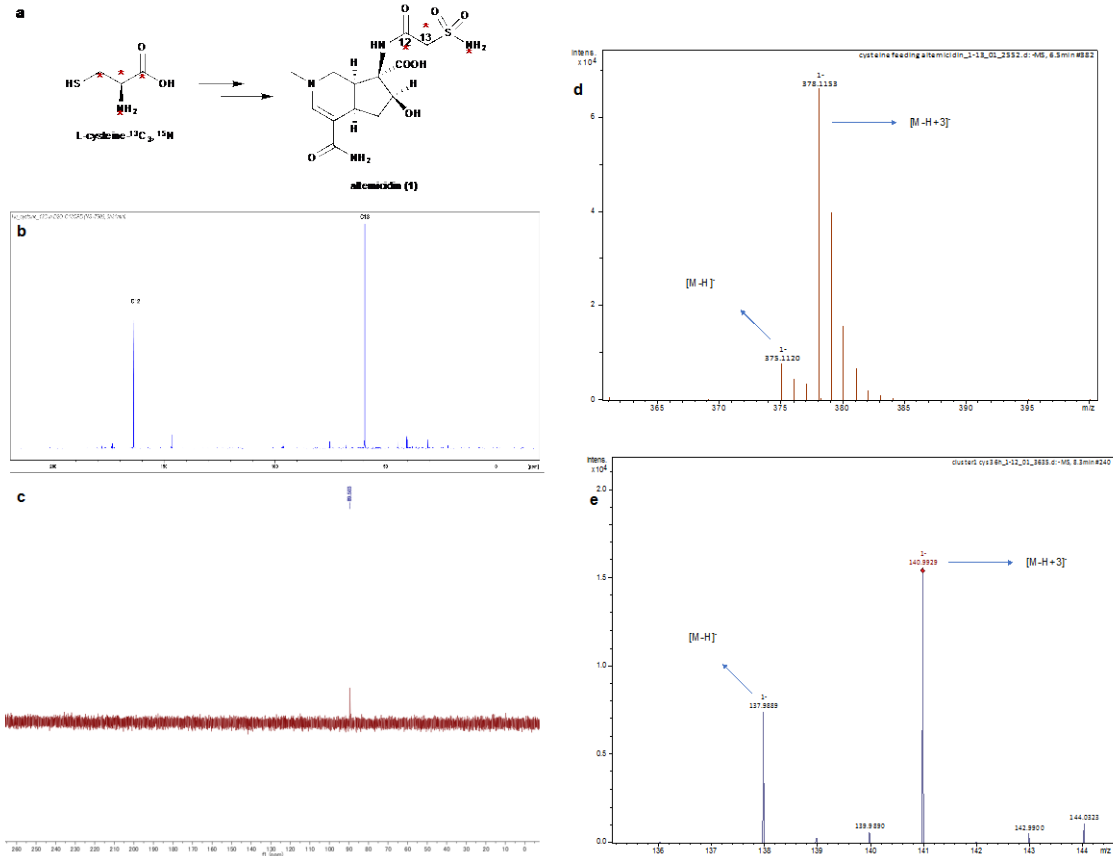

Supplementary Figure 16. L-cysteine-$^{13}C_3$, ^{15}N feeding results. **a**) L-cysteine-$^{13}C_3$, ^{15}N incorporated in altemicidin (**1**); **b**) ^{13}C NMR spectrum of the labeled altemicidin, C-12 and C-13 were labelled; **c**) ^{15}N NMR spectrum of the labeled altemicidin; **d**) MS spectrum of the labeled altemicidin (**1**); **e**) MS spectrum of the labeled 2-sulfamoylacetic acid (**5**).

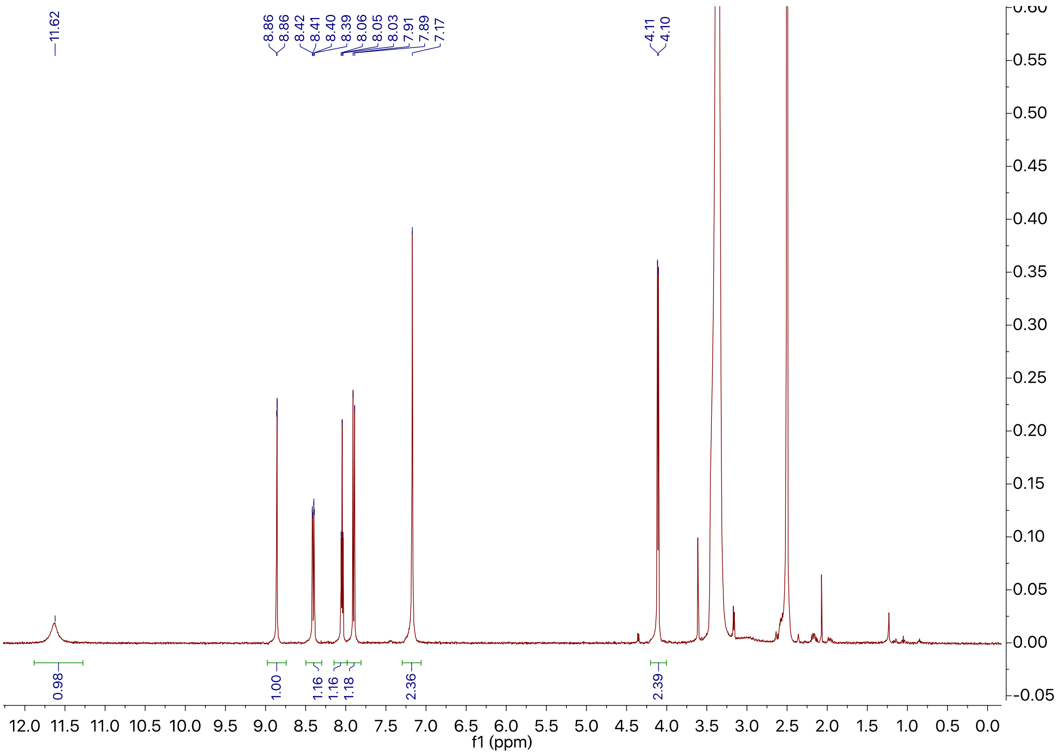

Supplementary Figure 17. ^{1}H NMR spectrum of DNPH-**7** (DMSO-*d6*)

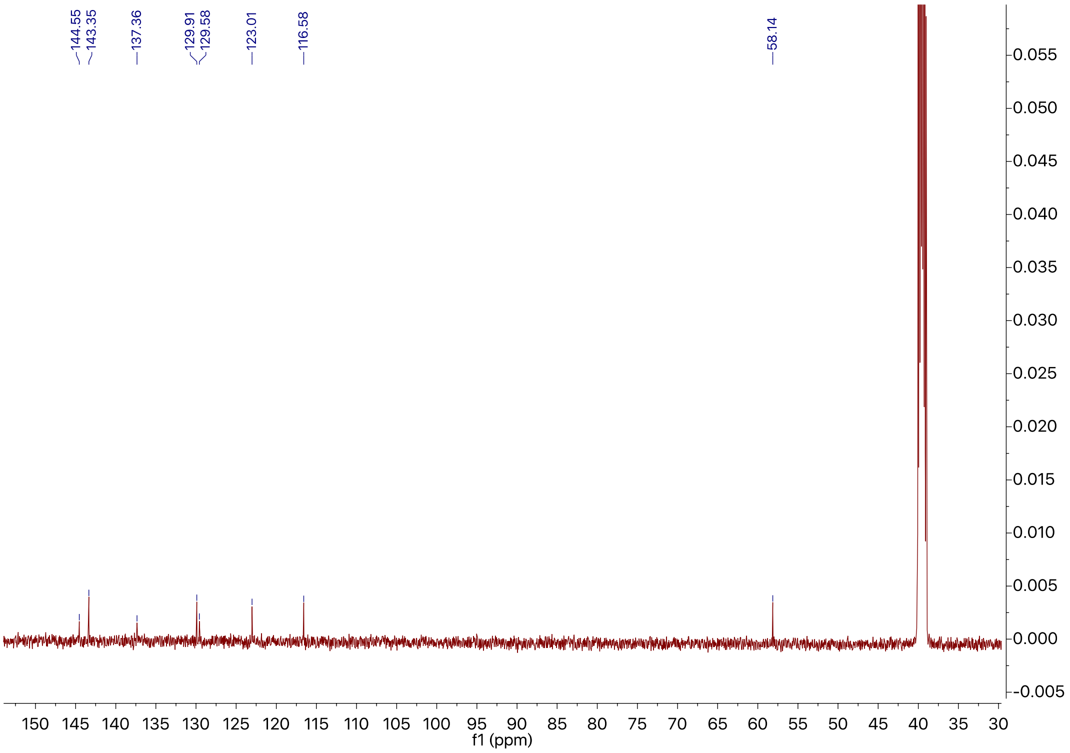

Supplementary Figure 18. ^{13}C NMR spectrum of DNPH-**7** (DMSO-*d6*)

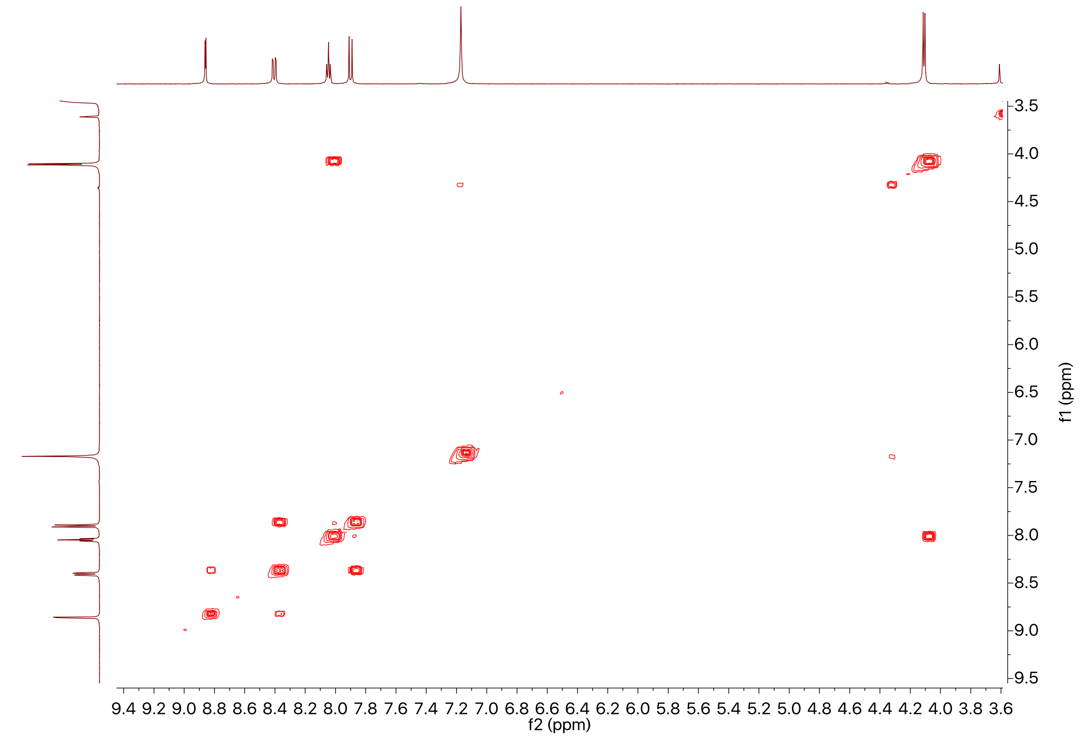

Supplementary Figure 19. COSY spectrum of DNPH-**7** (DMSO-*d6*)

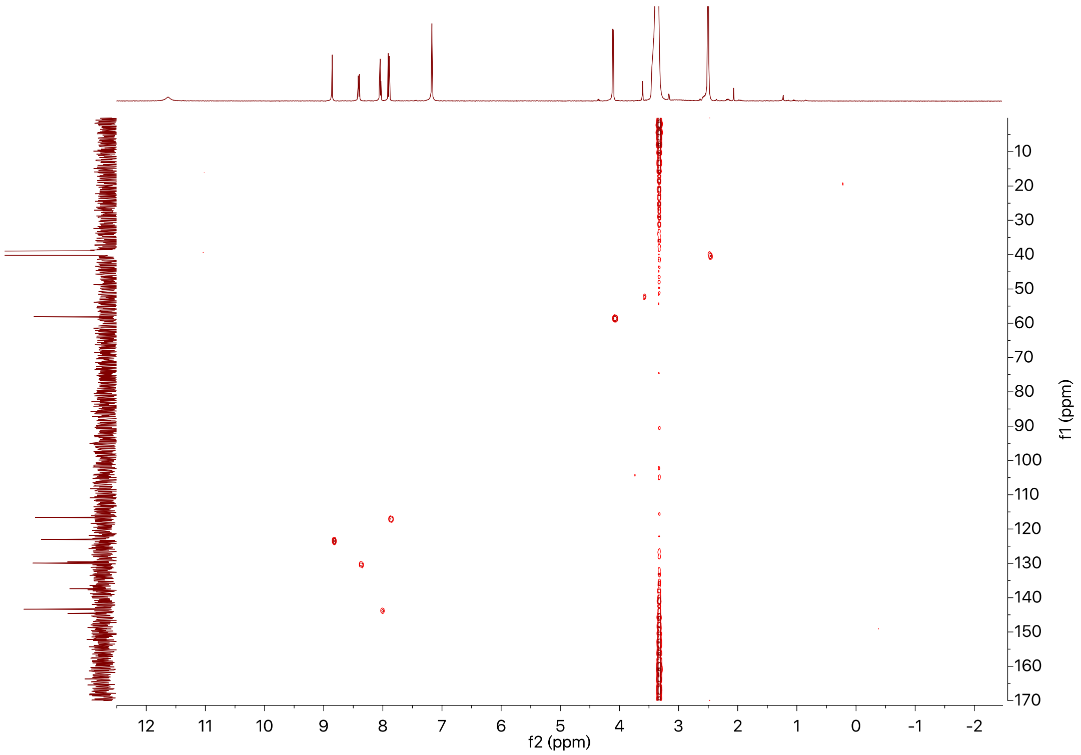

Supplementary Figure 20. HMQC spectrum of DNPH-**7** (DMSO-*d6*)

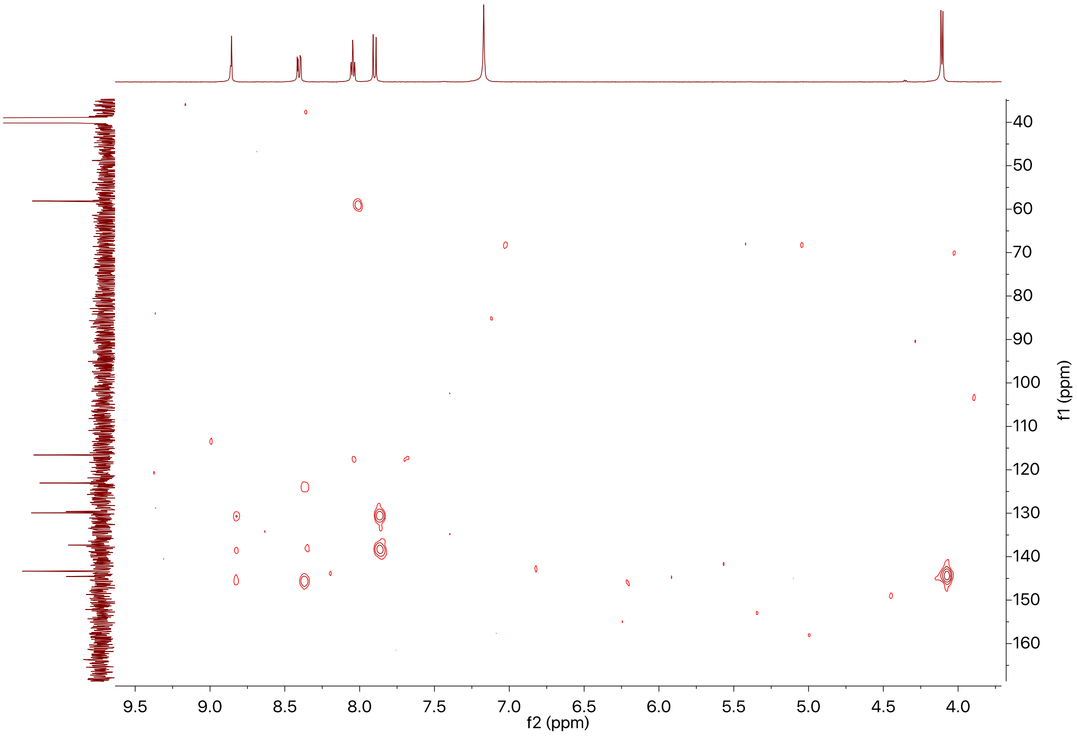

Supplementary Figure 21. HMBC spectrum of DNPH-**7** (DMSO-*d6*)

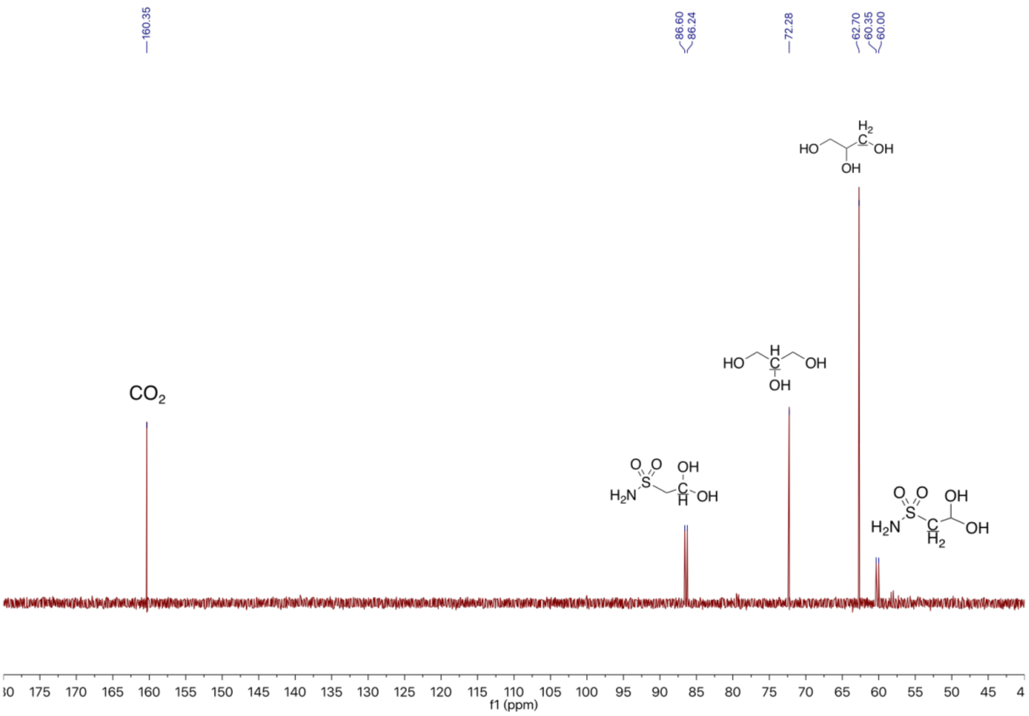

Supplementary Figure 22. ^{13}C NMR spectrum of the hydrated **7** generated from SbzM reaction with [$^{13}C_3$, $^{15}N_1$]-L-cysteine (D_2O). The reaction condition was same as the standard SbzM assay except the reaction time is 2 hr.

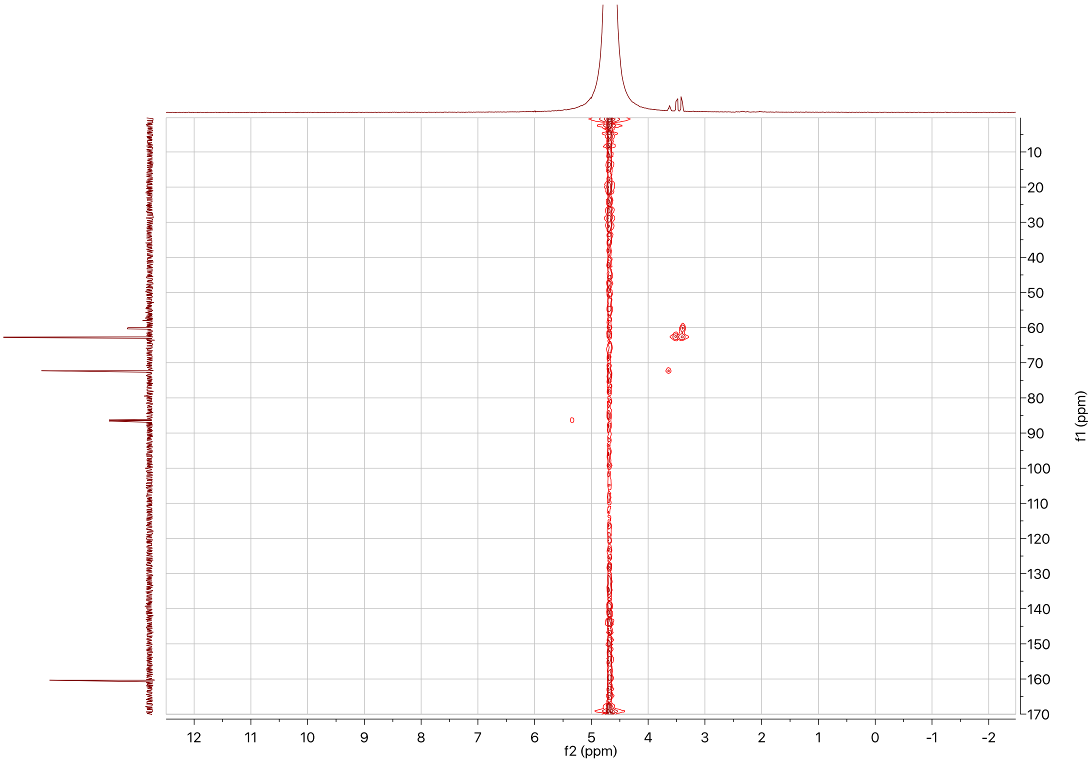

Supplementary Figure 23. HMQC spectrum of the hydrated **7** generated from SbzM reaction with $[^{13}C_3, ^{15}N_1]$-L-cysteine (D_2O). The chemical shift of H-1 and H-2 of the hydrated **7** was identified from this spectrum.

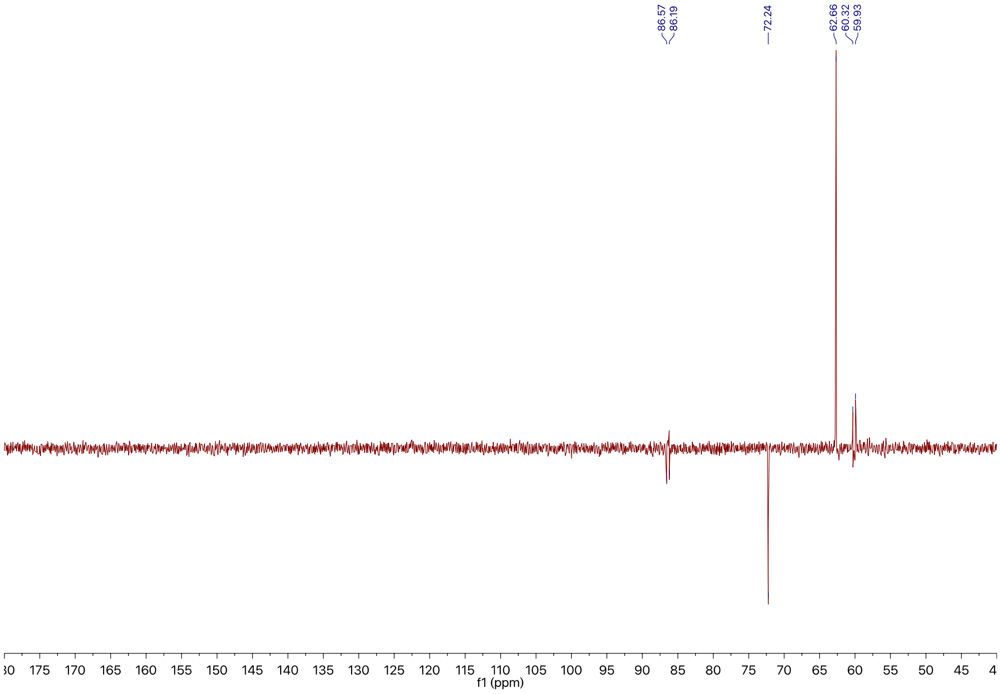

Supplementary Figure 24. DEPT135 spectrum of the hydrated **7** generated from SbzM reaction with $[^{13}C_3, ^{15}N_1]$-L-cysteine (D_2O).

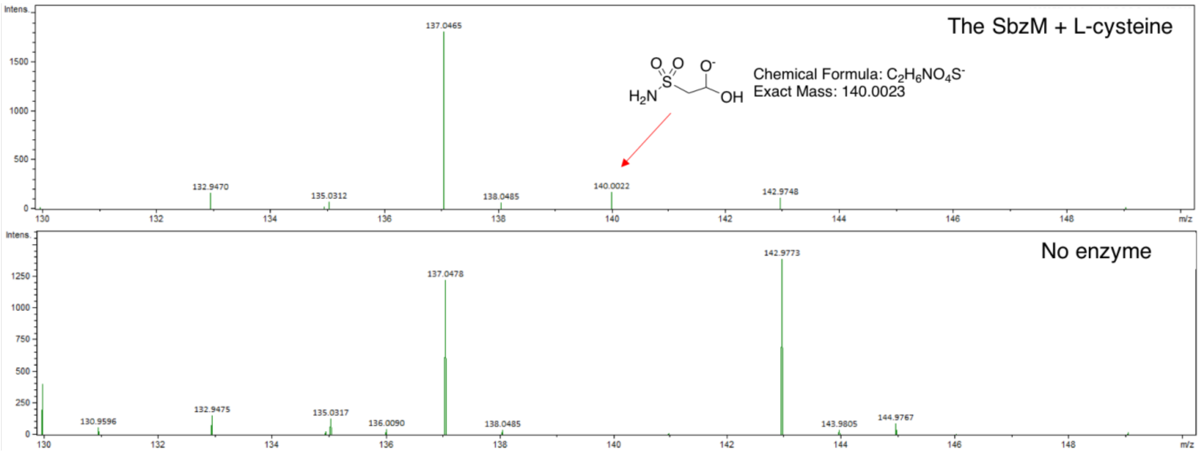

Supplementary Figure 25. HR-MS spectrum of the product of SbzM assay with L-cysteine. The assay was directly injected to Bruker Compact qTOF mass spectrometer with syringe pump (180 ml/hr), and the products were ionized with negative ion mode. The *m/z* 140.0022 which was not seen in the negative control (no enzyme) was assigned as the HR-MS of the hydrated **7**.

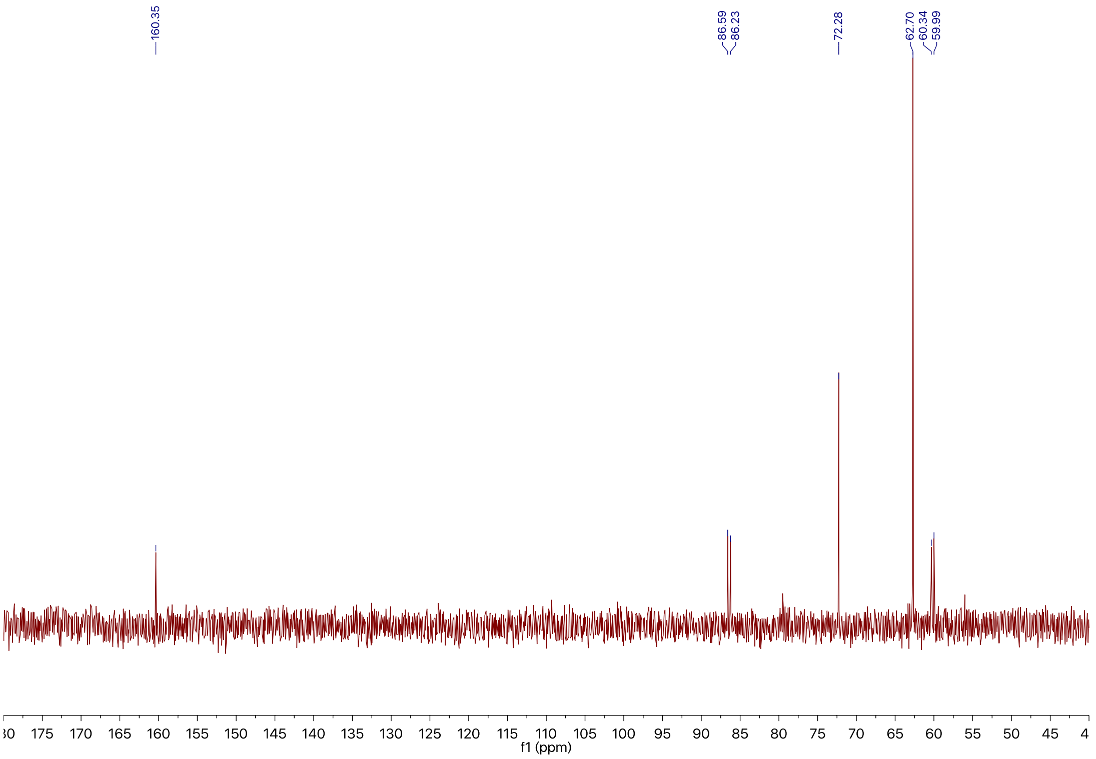

Supplementary Figure 26. ^{13}C NMR spectrum of the SbzM+SbzJ assay with [$^{13}C_3$, $^{15}N_1$]-L-cysteine (D_2O) in absence of NAD^+. SbzM was reacted with [$^{13}C_3$, $^{15}N_1$]-L-cysteine in 2 hr, and the reaction was further incubated in 30 min after SbzJ was added into the assay. The peaks in the spectrum do not differ from **Supplementary Figure 22.**

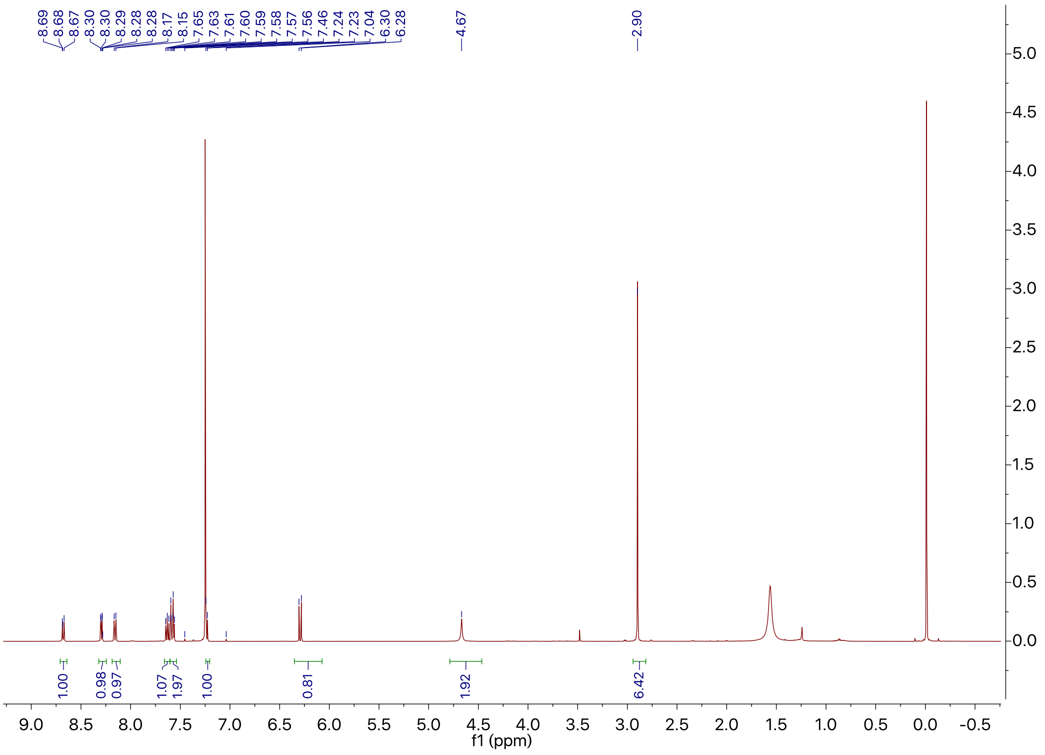

Supplementary Figure 27. ^{1}H NMR spectrum of DNS-**7b** ($CDCl_3$)

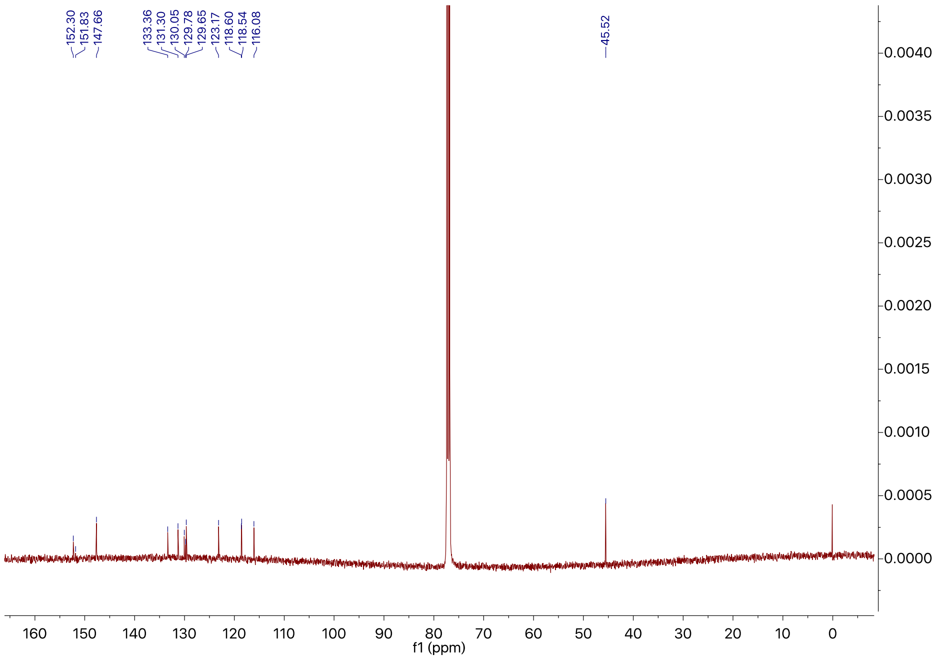

Supplementary Figure 28. ^{13}C NMR spectrum of DNS-**7b** ($CDCl_3$)

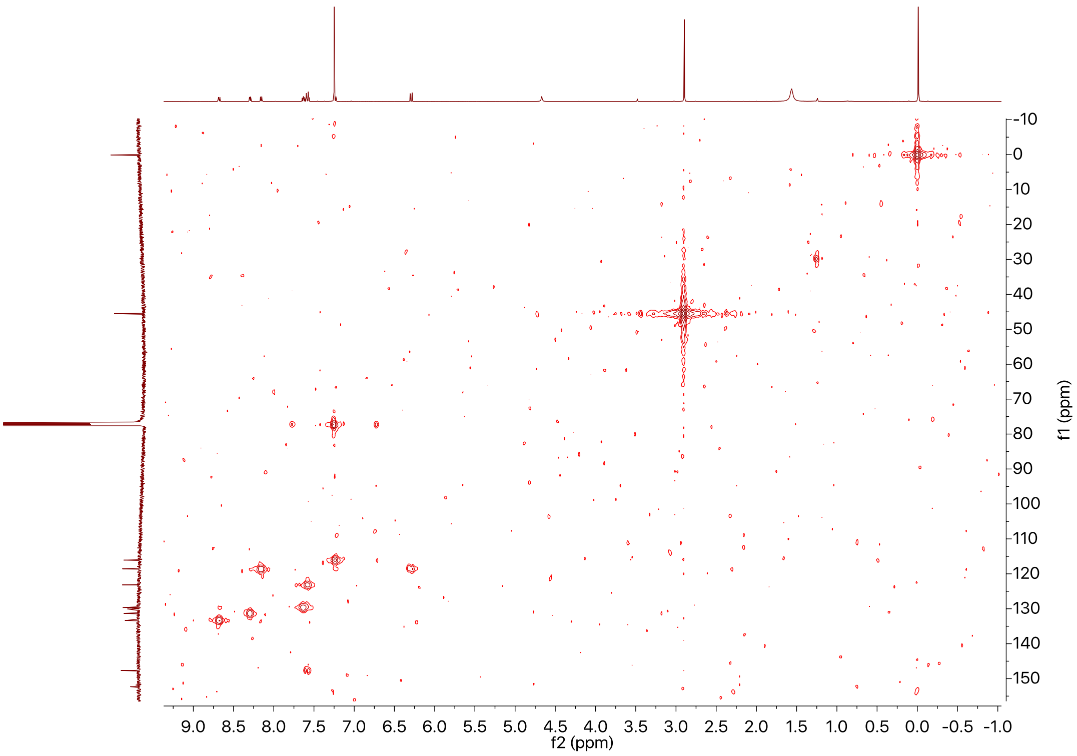

Supplementary Figure 29. HMQC spectra of DNS-**7b** ($CDCl_3$)

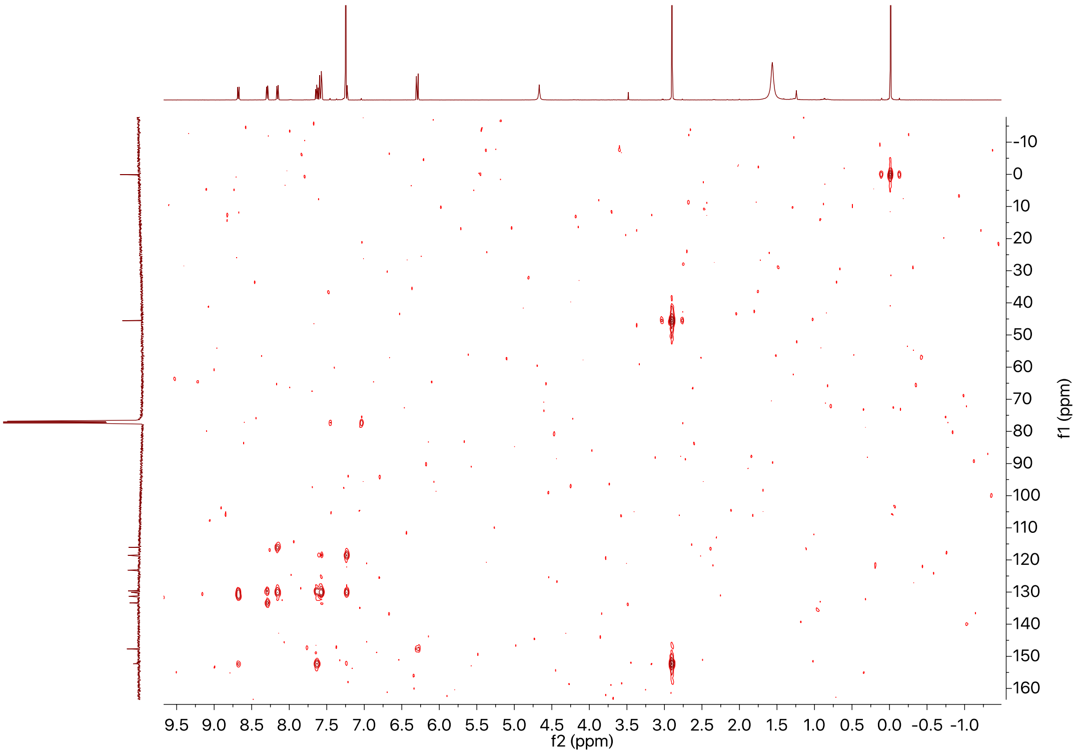

Supplementary Figure 30. HMBC spectra of DNS-**7b** ($CDCl_3$)

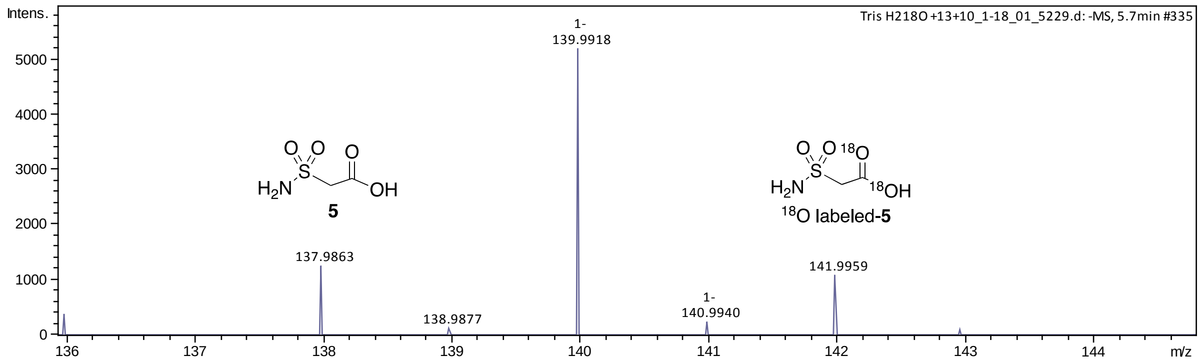

Supplementary Figure 31. MS spectra of the product of SbzM and SbzJ reaction in presence of L-cysteine and $H_2{}^{18}O$.

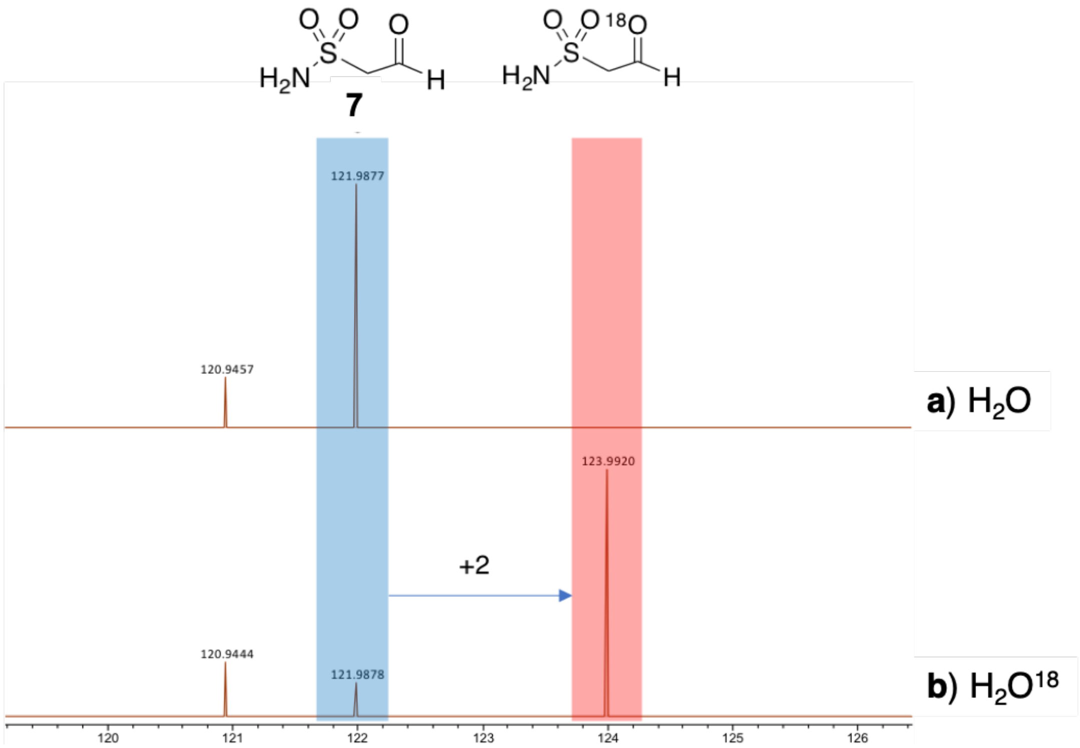

Supplementary Figure 32. The investigation of the SbzM reaction product in presence of L-cysteine with $H_2{}^{18}O$. The MS spectra of the product of SbzM reaction in presence of L-cysteine with (**b**) or without $H_2{}^{18}O$ (**a**). The LC-MS analysis was performed with HILIC pak VG-50 2D column (2.0 mm I.D.× 150 mm, Shodex) with a gradient from 80% CH_3CN-H_2O (50 mM ammonium acetate pH 8.0) to 10% in 20 min. Flow rate was 0.2 mL/min.

a

b

c

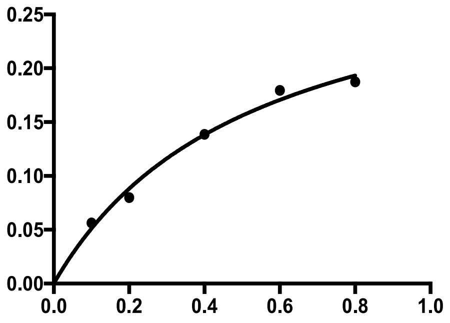

Supplementary Figure 33. Biochemical properties of SbzM. a) EIC of DNS-**7b** (m/z 357.0573); and b) relative activities; c) kinetic analysis by detection of the consumption of L-cysteine by LC-MS.

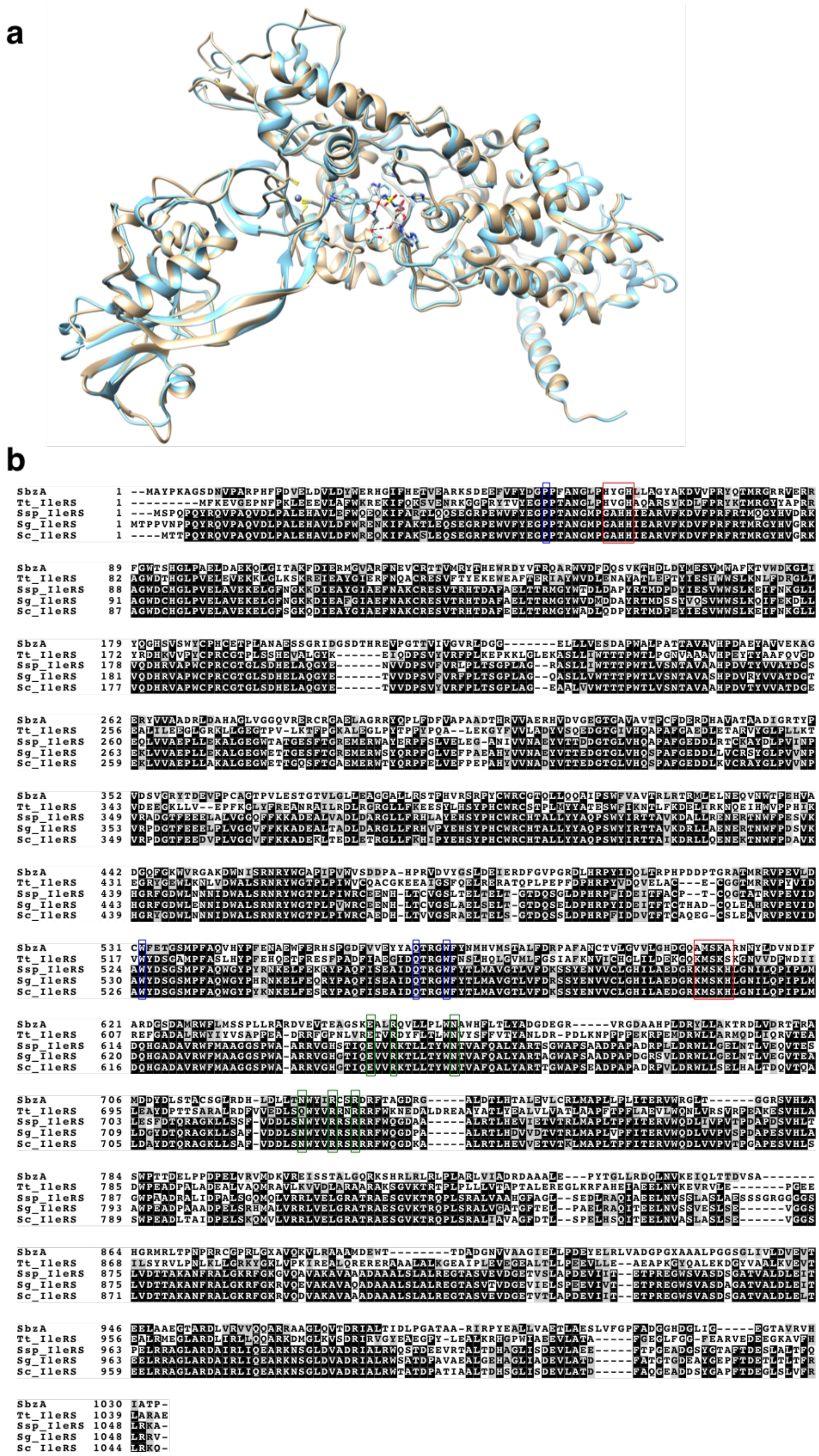

Supplementary Figure 34. The comparison between SbzA and the related proteins. (a) The homology model of SbzA. The homology model of SbzA (brown) was built based on the Ile-tRNA synthetase from *Thermus thermophilus* (Tt_IleRS) (1jzq.1.A cyan) in swiss-model server (https://swissmodel.expasy.org). The overlaid model was built by UCSF-Chimera. (b) The amino acid alignment among SbzA, Tt_IleRS, Ssp_IleRS, Sg_IleRS, and Sc_IleRS. The two well-known ATP-binding motif “HIGH” and “KMSKH” are in the red frames, the amino acids which supports isoleucyl moiety[1] are in the blue frames, and the amino acids to support tRNA are in the green frames.

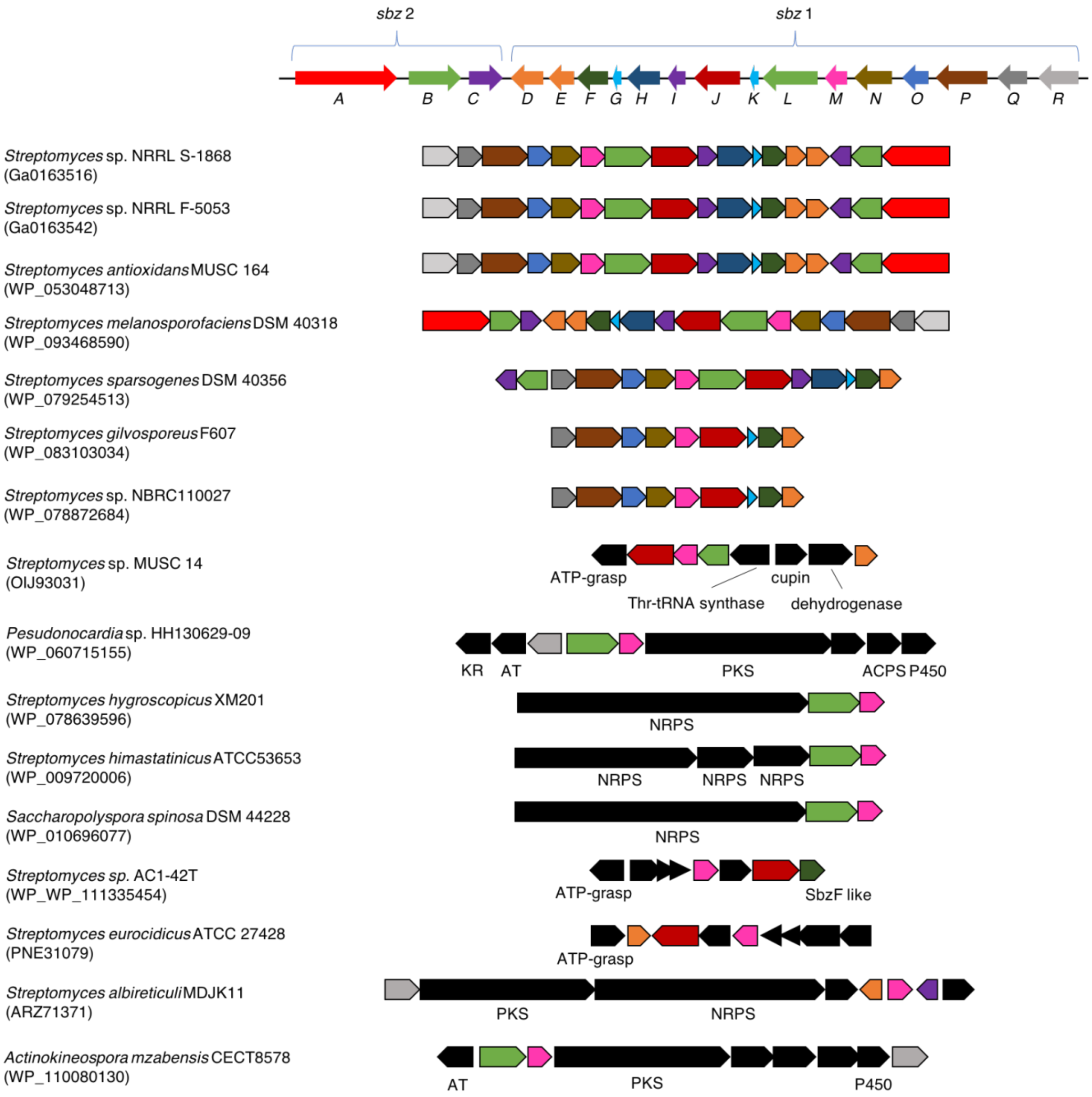

Supplementary Figure 35. Organization of gene clusters containing *sbzM* homologues. *sbzM* homologs were colored pink. The homologs of the other *sbz* genes are also depicted with the respective colors. The accession number of *sbzM* homologs are also depicted.

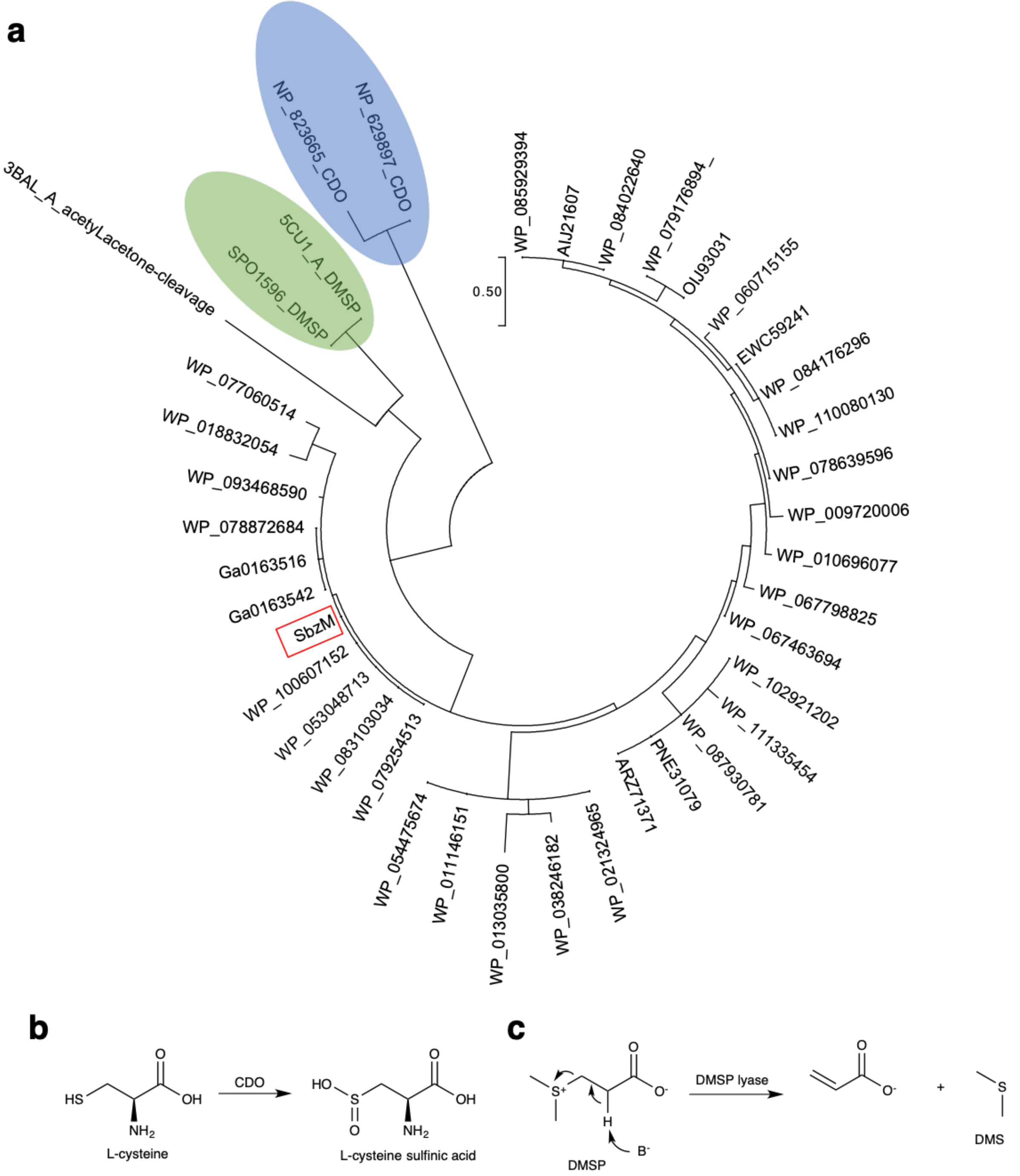

Supplementary Figure 36. The phylogenetic analysis of SbzM homologs. **a**) The phylogenetic tree of SbzM homologs (marked as red) . Cysteine dioxygenase (CDO) are marked as blue; DMSP lyase are marked as green. The alignment was done by Muscle alignment with default parameters, and the phylogenetic tree was constructed by maximum likelihood tree method with default parameters in MEGA7. **b**) enzyme reaction catalyzed by CDO, **c**) enzyme reaction catalyzed by DMSP lyase.

Supplementary Tables

Supplementary Table 1. Annotation of each protein in the *sbz* gene cluster

Gene	Amino acid (base pairs)	Closest protein homologue	Identity	Positive	Proposed function
sbzA	1033(3102)	Q5YYW9 (Nocardia farcinica IFM 10152)	57%	70%	Isoleucine-tRNA synthetase
sbzB	532(1599)	Q9R9J0 (Bacillus subtilis)	36%	53%	AMP-binding enzyme
sbzC	346(1041)	Q47LA0 (Thermobifida fusca YX)	41%	61%	GNAT family enzyme
sbzD	334(1005)	Q643C8 (Streptomyces hygroscopicus)	40%	56%	Methyltransferase MppJ like
sbzE	260(783)	P54458 (Bacillus subtilis subsp. subtilis str. 168)	27%	48%	Methyltransferase
sbzF	320(963)	E8NCH3 (Microbacterium testaceum StLB037)	30%	45%	F420-dependent glucose-6-phosphate dehydrogenase
sbzG	85(258)	Q89VT6 (Bradyrhizobium diazoefficiens USDA 110)	39%	63%	Peptidyl carrier protein
sbzH	334(1005)	P05149 (Acinetobacter calcoaceticus)	35%	53%	Aldose 1-epimerase
sbzI	188(567)	C7IZ16 (Oryza sativa Japonica Group)	28%	37%	GNAT family enzyme
sbzJ	464(1395)	Q6D6Y7 (Pectobacterium atrosepticum SCRI1043)	39%	52%	Gamma-aminobutyraldehyde dehydrogenase
sbzK	93(282)	A7ZA76 (Bacillus velezensis FZB42)	36%	49%	Peptidyl carrier protein
sbzL	559(1680)	O07610 (Bacillus subtilis subsp. subtilis str. 168)	28%	43%	AMP-binding enzyme
sbzM	228(687)	A1WGK0 (Verminephrobacter eiseniae EF01-2)	35%	45%	Cupin
sbzN	385(1158)	Q8TZ14 (Methanopyrus kandleri AV19)	27%	41%	Sugar isomerase
sbzO	268(807)	Q58515 (Methanocaldococcus jannaschii DSM 2661)	27%	51%	BtpA
sbzP	523(1572)	Q0S962 (Rhodococcus jostii RHA1)	34%	56%	Aminotransferase
sbzQ	304(915)	Q94FY7 (Arabidopsis thaliana)	38%	54%	Dioxygenase
sbzR	409(1230)	O34546 (Bacillus subtilis subsp.subtilis str.168)	24%	36%	MFS transporter

Supplementary Table 2. ^{1}H NMR and ^{13}C NMR data of DNPH-**7** (500 MHz, DMSO-*d6*)

	proton	carbon
1	-	144.6
2	-	129.6
3	8.86, d, *J* = 2.8 Hz	123.0
4	-	137.4
5	8.41, dd, *J* = 9.7, 2.8 Hz	129.9
6	7.90, d, *J* = 9.7 Hz	116.6
7	11.62, brs	-
9	8.05, t, *J* = 6.0 Hz	143.4
10	4.11, d, *J* = 6.0 Hz	58.1
12	7.17, s	-

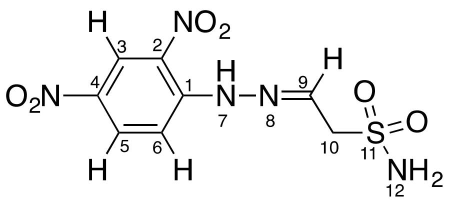

Supplementary Table 3. ^{1}H NMR and ^{13}C NMR data of DNS-**7b** (500 MHz, $CDCl_3$)

	proton	carbon
1	-	152.3
2	8.30 (d, *J* = 8.0 Hz)	131.3
3	7.64 (t, *J* = 8.0 Hz)	129.6
4	8.69 (d, *J* = 8.0 Hz)	133.4
4a	-	130.0
5	-	151.8
6	8.17 (d, *J* = 8.0 Hz)	118.6
7	7.58 (t, *J* = 8.0 Hz)	123.2
8	7.25 (d, *J* = 8.0 Hz)	116.1
8a	-	129.8
9	2.90 (s)	45.5
10	2.90 (s)	45.5
11	7.59 (d, *J* = 12 Hz)	147.7
12	6.30 (d, *J* = 12 Hz)	118.5
-NH	4.68 (brs)	-
-OH	4.68 (brs)	-

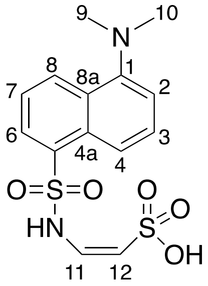

Supplementary References

1. Nakama, T., Nureki, O. & Yokoyama, S. Structural basis for the recognition of isoleucyl-adenylate and an antibiotic, mupirocin, by isoleucyl-tRNA synthetase. *J. Biol. Chem.* **276,** 47387–47393 (2001).
2. Taguchi, T. *et al.* Bifunctionality of ActIV as a cyclase-thioesterase revealed by in vitro reconstitution of actinorhodin biosynthesis in *Streptomyces coelicolor* A3(2). *ChemBioChem* **18,** 316-323 (2017).
