# Supplementary material for: Aminoacyl sulfonamide assembly in SB-203208 biosynthesis
Source: Nat Commun. 2019 Jan 14;10:184. doi: 10.1038/s41467-018-08093-x (PMC6331615; doi:10.1038/s41467-018-08093-x)
Supplement: Supplementary file 3 — Supplementary Data 1 [file 41467_2018_8093_MOESM3_ESM.docx]

| **Strains** | **Description** | **Source** |
| --- | --- | --- |
| *Streptomyces* sp. NCIMB 40513 | SB-203208 producer | GSK |
| *Streptomyces lividans* TK21 | Heterologous expression host | John Innes Centre |
| *S. lividans*/sbz1 | Heterologous expression | This study |
| *S. lividans*/sbz1&2 | Heterologous expression | This study |
|  |  |  |
| **Vectors** | **Description** | **Source** |
| pET28a | Vector for protein expression in *E. coli* | Novagen |
| pHSA81 | Vector for protein expression in *S. lividans* | Dr. M. Kobayashi |
| pZH1 | Vector for heterologous expression in *S. lividans* comprising $\phi$BT1, *aac(3)IV* and *ermE* promotor based on pUC19 | This study |
| pZH2 | Vector for heterologous expression in *S. lividans* comprising $\phi$C31, *tsr* and *ermE* promotor based on pUC19 | This study |
| pZH1-sbz1 | Vector for heterologous expression of cluster1 | This study |
| pZH2-sbz2 | Vector for heterologous expression of cluster2 | This study |
| pZH1-sbzR-G | Vectors for gene deletion of sbzF | This study |
| pZH2-sbzE-D |  | This study |
| pZH1-sbzR-I | Vectors for gene deletion of *sbzH* | This study |
| pZH2-sbzG-D |  | This study |
| pZH1-sbzR-K | Vectors for gene deletion of *sbzJ* | This study |
| pZH2-sbzI-D |  | This study |
| pZH1-sbzR-N | Vectors for gene deletion of *sbzM* | This study |
| pZH2-sbzL-D |  | This study |
| pZH1-sbzR-O | Vectors for gene deletion of *sbzN* | This study |
| pZH2-sbzM-D |  | This study |
| pZH1-sbzR-P | Vectors for gene deletion of *sbzO* | This study |
| pZH2-sbzN-D |  | This study |
| pZH1-sbzR-Q | Vectors for gene deletion of *sbzP* | This study |
| pZH2-sbzO-D |  | This study |
| pZH1-sbzR | Vectors for gene deletion of *sbzQ* | This study |
| pZH2-sbzP-D |  | This study |
| pET28a-sbzA | Vector for protein expression in *E. coli*. | This study |
| pET28a-sbzB | Vector for protein expression in *E. coli*. | This study |
| pET28a-sbzC | Vector for protein expression in *E. coli*. | This study |
| pET28a-sbzG | Vector for protein expression in *E. coli*. | This study |
| pET28a-sbzI | Vector for protein expression in *E. coli*. | This study |
| pET28a-sbzJ | Vector for protein expression in *E. coli*. | This study |
| pET28a-sbzK | Vector for protein expression in *E. coli*. | This study |
| pET28a-sbzL | Vector for protein expression in *E. coli*. | This study |
| pET28a-sbzM | Vector for protein expression in *E. coli*. | This study |
| pHSA81-sbzL | Vector for protein expression in *S. lividans.* | This study |
| pACYC-sfp | Vector for phosphopantetheinyl transferase in *E. coli*. | The former study^2^ |
| pET28a-Ssp_IleRS | Vector for protein expression in *E. coli*. | This study |
| **Primers** | **Sequence (5’**$\boldsymbol{\to}$ **3’)** | **Description** |
| Primer I | gacggccagtgaattcagctggggcgcagctcgaagag | Primers for amplification of sbz 1 |
| Primer II | gctgaacgccggcgaggtgtgggtc |  |
| Primer III | tcgccggcgttcagctcgccggccagttccagaaccgc |  |
| Primer IV | ccacgccgcgtgtgccgtgcagatgg |  |
| Primer V | gcacacgcggcgtggtaggagtcgccgtctcccacgatg |  |
| Primer VI | tgattacgccaagcttgtgctgaaccgaattctccccgcttc |  |
| Primer VII | aacgccagcaacgcggcctttttacggttcctggccttttgctg  gccttttggcgccggacggggcttcagacg | Primers for $\lambda$ RED recombination |
| Primer VIII | gccacggccagaacgcgtcccggtccggaagcggggaga  attcggttcagcatatgggtcctcctgtggag |  |
| Primer IX | cacaggaggacccatatggcatatccgaaagccgggtcc | Primers for amplification of sbz 2 |
| Primer X | gacggccagtgaattcgcaacactgctcgccttccagag |  |
| NdeI-Orf7R | tgagagtgcaccatatgtcagcccgactggtggcggcac | Primers for gene deletion of *sbzF* |
| NdeI-Orf5F | cacaggaggacccatatggggaactattactacacggacg |  |
| NdeI-Orf9R | tgagagtgcaccatatggatcgtcgctgccatcagtcctc | Primers for gene deletion of *sbzH* |
| NdeI-Orf7F | cacaggaggacccatatggaccccctcatggcttccgtg |  |
| NdeI-Orf11R | tgagagtgcaccatatgcatggcaggggggccgctcac | Primers for gene deletion of *sbzJ* |
| NdeI-Orf9F | cacaggaggacccatatggcggacacccgcattccgg |  |
| NdeI-Orf14R | tgagagtgcaccatatgtcatggcgtctctccgtcgtgtc | Primers for gene deletion of *sbzM* |
| NdeI-Orf12F | cacaggaggacccatatggtctcttcgtcccccaccgac |  |
| NdeI-Orf15R | tgagagtgcaccatatgctcatcgctccagcctccgtacg | Primers for gene deletion of *sbzN* |
| NdeI-Orf13F | cacaggaggacccatatgatcgagacgacagagggcatg |  |
| NdeI-Orf16R | tgagagtgcaccatatgcatgggtcagctccccgtcgtg | Primers for gene deletion of *sbzO* |
| NdeI-Orf14F | cacaggaggacccatatgagcgacgggaacggcccgg |  |
| NdeI-Orf17R | tgagagtgcaccatatggcctagacgatgcgcagacgcttg | Primers for gene deletion of *sbzP* |
| NdeI-Orf15F | cacaggaggacccatatggacggatggacggcccctg |  |
| NdeI-Orf18R | tgagagtgcaccatatgtcagccgcagtgcgccggacgtac | Primers for gene deletion of *sbzQ* |
| NdeI-Orf16F | cacaggaggacccatatgaaactcttcgaatcccttccgtc |  |
| Orf1-NdeI-f | gcgcggcagccatatggcatatccgaaagccgggtcc | Primers for sbzA expression |
| Orf1-HindIII-r | gtgcggccgcaagcttcagggcgtggcgatgtgcac |  |
| Orf2-NdeI-f | cgcgcggcagccatatgacgcgcctcgaagggcttg | Primers for sbzB expression |
| Orf2-HindIII-r | gtgcggccgcaagcttggtgagggagaacccggtcacg |  |
| Orf3-NdeI-f | gcgcggcagccatatgaccgggttctccctcaccg | Primers for sbzC expression |
| Orf3-HindIII-r | gtgcggccgcaagcttcatcgccacgcgcggccggac |  |
| Orf7-NdeI-f | cgcgcggcagccatatggaccccctcatggcttcc | Primers for sbzG expression |
| Orf7-HindIII-r | gtgcggccgcaagcttggggttcagcccgactggtgg |  |
| Orf9-NdeI-f | cgcgcggcagccatatggcggacacccgcattccgg | Primers for sbzI expression |
| Orf9-HindIII-r | gtgcggccgcaagcttccggatcgtcgctgccatcagtc |  |
| Orf10-NdeI-f | cgcgcggcagccatatgccccaggagcgacagaagcg | Primers for sbzJ expression |
| Orf10-HindIII-r | gtgcggccgcaagcttcagctcccgcccagacgtacg |  |
| Orf11-NdeI-f | cgcgcggcagccatatgaacgacccctccgggatgac | Primers for sbzK expression |
| Orf11-HindIII-r | gtgcggccgcaagcttctgtcgctcctggggcatggcagg |  |
| Orf12-NdeI-f | cgcgcggcagccatatggtctcttcgtcccccaccgac | Primers for sbzL expression |
| Orf12-HindIII-r | gtgcggccgcaagcttcatcgggtgctcccggagttc |  |
| Orf13-NdeI-f | cgcgcggcagccatatgtccacgcagcaggtcatcgc | Primers for sbzM expression |
| Orf13-HindIII-r | gtgcggccgcaagcttcacccgaaccggtggggggagtgc |  |
| pET28a-pHSA81NdeIf | aaaggaatgagcatatgggcagcagccatcatcatc | Primers for cloning from pET28a to pHSA81 |
| pET28a-pHSA81HindIIIr | tagtgctagcaagctgctcgagtgcggccgcaagc |  |
| Ssp_IleRS-NdeI-f | cgcgcggcagccatatgagtccgcagccccagtaccg | Primers for Ssp_IleRS expression |
| Ssp_IleRS-HindIII-r | gtgcggccgcaagcttacgccttgcgcagctggaacgtcag | Primers for Ssp_IleRS expression |
